# Red Light‐Triggered Anti‐Angiogenic and Photodynamic Combination Therapy of Age‐Related Macular Degeneration

**DOI:** 10.1002/advs.202301985

**Published:** 2023-09-14

**Authors:** Shuting Xu, Kaixuan Cui, Kaiqi Long, Jia Li, Ni Fan, Wai‐Ching Lam, Xiaoling Liang, Weiping Wang

**Affiliations:** ^1^ State Key Laboratory of Pharmaceutical Biotechnology Department of Pharmacology and Pharmacy Li Ka Shing Faculty of Medicine Laboratory of Molecular Engineering and Nanomedicine Dr. Li Dak‐Sum Research Centre The University of Hong Kong Hong Kong SAR China; ^2^ State Key Laboratory of Ophthalmology Zhongshan Ophthalmic Center Guangdong Provincial Key Laboratory of Ophthalmology and Visual Science Sun Yat‐sen University Guangzhou 510060 China; ^3^ Department of Ophthalmology Vancouver General Hospital Vancouver BC V5Z 0A6 Canada

**Keywords:** age‐related macular degeneration, dasatinib, dimeric prodrug, photodynamic therapy, red‐light activation

## Abstract

Choroidal neovascularization (CNV) is the key pathological event of wet age‐related macular degeneration (wAMD) leading to irreversible vision loss. Currently, anti‐angiogenic therapy with anti‐vascular endothelial growth factor (VEGF) agents has become the standard treatment for wAMD, while it is still subject to several limitations, including the safety concerns of monthly intravitreal administration and insufficient efficacy for neovascular occlusion. Combined therapy with photodynamic therapy (PDT) and anti‐angiogenic agents has emerged as a novel treatment paradigm. Herein, a novel and less‐invasive approach is reported to achieve anti‐angiogenic and photodynamic combination therapy of wAMD by intravenous administration of a photoactivatable nanosystem (Di‐DAS‐VER NPs). The nanosystem is self‐assembled by reactive oxygen species (ROS)‐sensitive dasatinib (DAS) prodrug and photosensitizer verteporfin (VER). After red‐light irradiation to the diseased eyes, intraocular release of anti‐angiogenic DAS is observed, together with selective neo‐vessels occlusion by VER‐generated ROS. Notably, Di‐DAS‐VER NPs demonstrates promising therapeutic efficacy against CNV with minimized systemic toxicity. The study enables an efficient intravenous wAMD therapy by integrating a photoactivation process with combinational therapeutics into one simple nanosystem.

## Graphical abstract

A red‐light‐triggered intraocular drug release strategy is applied to achieve an anti‐angiogenic and photodynamic combination treatment of wet age‐related macular degeneration (wAMD) via intravenous administration of a photoactivatable nanosystem (Di‐DAS‐VER NPs). The nanosystem demonstrates strengthened choroidal neovascularization suppression and favorable biosafety profiles in vivo. This novel design has clinical potential for minimally‐invasive delivery of combination therapeutics for treating angiogenic eye diseases.

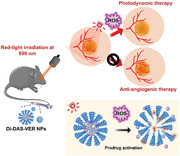

## 1 Introduction

Age‐related macular degeneration (AMD) has become the leading cause of irreversible vision impairment among the elderly worldwide.^[^ ^1^, ^2^ ^]^ Although the wet (exudative) form of AMD (wAMD) only constitutes a small proportion (≈10%) of AMD cases, it accounts for the majority of severe vision impairment. wAMD is characterized by the progressive development of choroidal neovascularization (CNV) into subretinal space and concomitant retinal damage.^[^ ^3^, ^4^ ^]^ The current treatments for delaying wAMD progression include anti‐angiogenic therapy, photodynamic therapy (PDT), and thermal laser treatment, etc.^[^ ^5^ ^]^ Remarkably, given the critical roles of the upregulated vascular endothelial growth factor (VEGF) level in CNV growth, anti‐angiogenetic therapy has been regarded as the first‐line treatment for wAMD.^[^ ^5^ ^]^ However, the established vessels are protected by developed pericytes and less sensitive to anti‐angiogenetic agents,^[^ ^6^, ^7^ ^]^ which limits the efficacy of sole anti‐angiogenetic treatment to destroy the abnormally grown vessels in wAMD eyes.

PDT with verteporfin (VER) is a clinically available option for vascular occlusion of CNV, especially for polypoidal choroidal vasculopathy (PCV), a subtype of wAMD that is more frequently observed in the Asian population.^[^ ^8^, ^9^ ^]^ PDT can selectively damage the vascular endothelium and obliterate polypoidal lesions through singlet oxygen production, leading to local thrombosis and CNV closure.^[^ ^10^ ^]^ However, it was reported that PDT could lead to compensatory upregulation of angiogenetic factors like VEGF, which compromised its long‐term effectiveness toward wAMD.^[^ ^11^ ^]^ Therefore, combining anti‐angiogenetic therapy and PDT would be desirable for the long‐term management of wAMD.^[^ ^7^ ^]^ Recently, clinical studies have demonstrated encouraging outcomes of intravitreal administration of anti‐angiogenic and PDT agents regarding visual acuity improvement, exudation reduction, and lower incidence of adverse events for wAMD patients.^[^ ^11^, ^12^, ^13^ ^]^ Thus, the strategy of combining anti‐angiogenic therapy and PDT should provide more efficient approaches to wAMD treatment.

**Figure 1:**
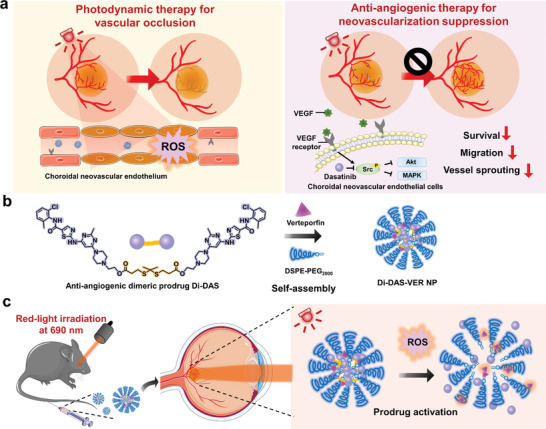
Schematic illustration of the photoactivatable prodrug‐based nanosystem (Di‐DAS‐VER NPs) for anti‐angiogenic and photodynamic combination therapy of wet age‐related macular degeneration (wAMD). a) Schematic representation of therapeutic mechanisms of photodynamic therapy (PDT) and anti‐angiogenic therapy for wAMD treatment. PDT directly causes vessel occlusion by reactive oxygen species (ROS)‐induced endothelial cell death and thrombosis. On the other hand, anti‐angiogenic therapy suppresses choroidal neovascularization (CNV) by inhibiting vascular endothelial growth factor (VEGF)‐related pathways. b) Schematic representation of self‐assembly of Di‐DAS‐VER NPs by anti‐angiogenic dimeric prodrug Di‐DAS, photosensitizer verteporfin, and amphiphilic lipid DSPE‐PEG_2000_. c) Schematic illustration showing the treatment process with Di‐DAS‐VER NPs. In a laser‐induced CNV mouse model, after intravenous injection of Di‐DAS‐VER NPs and applying red light at 690 nm to the diseased eye, VER‐induced ROS would trigger the cleavage of the prodrug and result in the intraocular release of anti‐angiogenic dasatinib. DAS: dasatinib. VER: verteporfin.

Furthermore, safety concerns remain regarding the monthly intravitreal injections of therapeutic agents into the vitreous body. Such invasive intraocular administration method usually causes mental stress and low patient compliance, and even intraocular adverse events such as endophthalmitis, traumatic cataract, vitreous hemorrhage, etc.^[^ ^14^ ^]^ Less‐invasive drug administration routes, like intravenous injection, can minimize the risks of intraocular adverse events, whereas systemic exposure of anti‐angiogenic agents increases the risks of systemic side effects like cardiovascular events and kidney injury.^[^ ^15^, ^16^ ^]^ Thus, it would be highly desirable to non‐invasively deliver therapeutic agents into eyes with minimal systemic side effects for wAMD treatment. In this study, for the first time, we developed a strategy to trigger anti‐angiogenic and PDT combination therapy in wAMD lesions by intravenously administrating a photoactivable nanosystem and irradiating the diseased eyes (**Figure** **1**). To illustrate, the photoactivatable prodrug‐based nanoparticles, Di‐DAS‐VER NPs, were developed by co‐assembly of a ROS‐responsive dimeric dasatinib prodrug Di‐DAS, an FDA‐approved photosensitizer verteporfin (VER), and amphiphilic lipid DSPE‐PEG_2000_. Upon 690 nm red‐light irradiation, verteporfin generates ROS to have the PDT effect and meanwhile triggers the cleavage of the dimeric prodrug, followed by a cascaded dasatinib (DAS) release. In a laser‐induced CNV mouse model, intravenous administration of such a prodrug‐based nanosystem followed by red‐light irradiation on wAMD eyes led to ROS production and intraocular DAS release, enabling PDT‐induced vascular occlusion and DAS‐induced CNV suppression. Notably, systemic side effects through intravenous administration are minimized due to the inactivity of the prodrug without red‐light exposure in the normal tissues.

## 2 Results

### 2.1 Preparation and Characterization of the Prodrug‐Based Nanoparticles

Regarding that Src kinase activity is required for VEGF‐mediated angiogenesis,^[^ ^17^, ^18^ ^]^ DAS, an Src family kinase inhibitor with a terminal hydroxyl group,^[^ ^19^ ^]^ was chosen for the prodrug design. The dimeric prodrug (Di‐DAS) was synthesized by bridging two DAS molecules with a ROS‐cleavable thioketal (TK) linker via the esterification reaction (Figure S1, Supporting Information). Proton nuclear magnetic resonance (^1^H NMR) and mass spectra confirmed the successful synthesis of Di‐DAS prodrug (Figure S2, Supporting Information).

**Figure 2:**
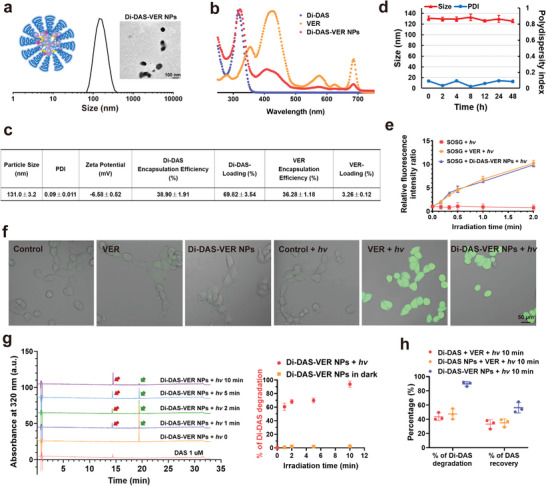
Characterization of Di‐DAS‐VER NPs. a) Hydrodynamic size distribution and transmission electron microscope image of Di‐DAS‐VER NPs. b) UV–Vis absorption spectra of free Di‐DAS, verteporfin (VER) and Di‐DAS‐VER NPs. c). Physiochemical properties of Di‐DAS‐VER NPs. d) Dynamic light scattering data of Di‐DAS‐VER NPs dispersed in PBS (pH 7.4) at 37°C for 48 h. e) Relative fluorescence intensity ratio of different formulations exposed to 690 nm laser irradiation with Singlet Oxygen Sensor Green (SOSG) as the singlet oxygen detection probe. The fluorescence intensity ratio was calculated as the fluorescence intensity at the prescribed time point versus that of the initial fluorescence intensity. Ex/Em = 488/525 nm. f) Intracellular ROS generation by free verteporfin and Di‐DAS‐VER NPs with or without light irradiation. HUVECs were treated with free verteporfin or Di‐DAS‐VER NPs (0.1 µm verteporfin) for 4 h, followed by incubation with 20 µm DCFH‐DA, and 690 nm light irradiation (10 mW cm^−2^, 400 s) or not. g) Representative HPLC chromatograms of Di‐DAS‐VER NPs upon 690 nm laser irradiation (100 mW cm^−2^) and quantitative analysis of Di‐DAS degradation rates for Di‐DAS‐VER NPs with or without light exposure. Red arrows denote the generated DAS while green arrows denote the decreased Di‐DAS. h) Quantitative analysis of Di‐DAS degradation rates and DAS recovery percentage for different formulations upon 690 nm light irradiation (100 mW cm^−2^, 10 min). Data were presented as mean ± standard deviations. *n* = 3.

Since hydrophobic dimeric conjugates tend to assemble into nanoparticles in an aqueous solution owing to strong intermolecular force,^[^ ^20^ ^]^ we investigated the self‐assembly behavior of hydrophobic Di‐DAS prodrug. We found that the incorporation of DSPE‐PEG_2000_ and verteporfin, a clinically‐available hydrophobic photosensitizer for wAMD treatment, facilitated the formation of photoresponsive nanoassemblies. We subsequently prepared the prodrug‐based nanoparticles by the nanoprecipitation method with the optimized feeding ratios of 1/15 (verteporfin: Di‐DAS, wt./wt.) and 1/5 (DSPE‐PEG_2000_: Di‐DAS, wt./wt.) according to size distribution and VER loading capacity of the formulations (Figure S3, Supporting Information). The hydrodynamic diameter of the prepared nanoparticles was ≈130 nm, with a relatively low polydispersity index (PDI) of 0.15 and negative surface zeta potential of ≈−6.58 mV (**Figure** **2**a). The spherical morphology and narrow size distribution of Di‐DAS‐VER NPs were further confirmed by transmission electron microscopy (TEM) image (Figure 2a). Additionally, the nanoparticle solution exhibited characteristic absorption peaks of Di‐DAS and verteporfin at 320 nm and 690 nm, respectively (Figure 2b), ascribing to successful co‐assembly of the two molecules. The loading capacity was 69.82% for Di‐DAS and 3.26% for verteporfin (Figure 2c). Moreover, there was no dramatic size change of the nanoparticles stored in PBS or DMEM containing 10% fetal bovine serum (FBS) at 37 °C for 48 h (Figure 2d; Figure S4, Supporting Information), confirming its favored colloidal stability in biological buffers. These results demonstrate that thioketal bond‐linked dimeric prodrug Di‐DAS can form stable and well‐dispersed nanoparticles with verteporfin, which can serve as a photoactivable nanosystem for ocular drug delivery.

### 2.2 Evaluation of ROS Generation by the Nanoparticles Upon Red‐Light Irradiation

Since ROS produced by verteporfin is responsible for vascular occlusion and simultaneous cleavage of the anti‐angiogenic prodrug Di‐DAS, we first monitored the singlet oxygen (^1^O_2_) production by Di‐DAS‐VER NPs under 690 nm laser irradiation (100 mW cm^−2^) using singlet oxygen sensor green (SOSG). SOSG emits weak blue fluorescence initially while possessing strong green fluorescence with the maximum wavelength of 525 nm after reaction with ^1^O_2_.^[^ ^21^ ^]^ As shown in Figure 2e, a time‐dependent increase in SOSG fluorescence intensity at 525 nm could be observed in the Di‐DAS‐VER NPs plus light irradiation group. The intensity is similar to that of the verteporfin plus light irradiation group, indicating that ^1^O_2_ generation ability of Di‐DAS‐VER NPs was comparable to that of free verteporfin. Furthermore, we also examined the in vitro ROS generation ability of the nanoparticles using 2′,7′‐dichlorfluorescein diacetate (DCFH‐DA) probe. Human umbilical vein endothelial cells (HUVECs), which commonly serve as in vitro endothelial cell model of CNV,^[^ ^22^ ^]^ were used for in vitro evaluations. We found the most significant increase in HUVEC uptake of Di‐DAS‐VER NPs at 4 h post‐incubation and thereafter applied 4 h of incubation for in vitro evaluations (Figure S5, Supporting Information). As shown in Figure 2f, little DCF fluorescence signal could be observed within HUVECs after exposure to light irradiation only, or after treatment with different formulations without light exposure. As a comparison, cells treated with Di‐DAS‐VER NPs plus light irradiation exhibited strong DCF fluorescence, which validates that Di‐DAS‐VER NPs can efficiently generate ROS upon red‐light irradiation in vitro.

### 2.3 Characterization of Red Light‐Triggered Drug Release Profiles

The thioketal linkage incorporated in the prodrugs can be cleaved upon ROS generation and subsequently trigger the release of parent drugs.^[^ ^21^, ^23^ ^]^ Thus, we investigated whether red light‐activatable ROS production could trigger the cleavage of the dimeric prodrugs and the cascade release of the active anti‐angiogenic agent, DAS. Di‐DAS‐VER NP solution was irradiated with a 690 nm laser (100 mW cm^−2^) for different time periods before high performance liquid chromatography (HPLC) analysis. As shown in Figure 2g, the peak at 19.4 min, corresponding to Di‐DAS, decreased dramatically, while the new peak at 14.2 min, corresponding to free DAS, emerged and gradually increased upon exposure to red‐light irradiation. In contrast, almost no Di‐DAS was degraded and no DAS was generated during the same periods without light irradiation (Figure S6, Supporting Information). When prolonging the irradiation period to 10 min, the degradation of Di‐DAS reached nearly 92%. In comparison, negligible Di‐DAS was consumed in the dark (Figure 2g). Meanwhile, we also detected the red light‐responsive DAS release from the aqueous solution of Di‐DAS plus VER and the aqueous solution of Di‐DAS nanoparticles plus VER after 10 min of 690 nm laser irradiation. As shown in Figure 2h, both formulations showed incomplete elimination of the prodrug within 10 min period of the light irradiation. The DAS recovery rates were ≈34% for these two groups, only 0.6‐fold relative to that of Di‐DAS‐VER NPs under the same irradiation condition (56.3% of DAS recovery). The discrepancy in their photoresponsive drug release profiles might be explained by the short lifetime and limited diffusion distance of ROS in aqueous solutions.^[^ ^24^ ^]^ Therefore, the assembly of Di‐DAS and the photosensitizer greatly facilitated the ROS‐triggered lysis process of Di‐DAS compared with another two formulations where verteporfin and Di‐DAS were separately dispersed in aqueous solutions. Overall, these results clearly demonstrate that the prodrug‐based nanoparticles can be activated and efficiently generate free DAS in response to red‐light irradiation.

In addition, we also investigated DAS release from Di‐DAS‐VER NPs at normal or pathological ROS levels. In vitro release experiments were performed in the presence of 1 µm H_2_O_2_ and 1 mm H_2_O_2_ according to the literature.^[^ ^25^, ^26^, ^27^ ^]^ As shown in Figure S7 (Supporting Information), DAS recovery percentages were quite low under both conditions, about 9.45% and 11.90% at 48 h post‐incubation with 1 µm H_2_O_2_ or 1 mm H_2_O_2_, far less than that of Di‐DAS‐VER NPs plus light irradiation (56.3% of DAS recovery). These data suggested that ROS produced by activated VER might be more effective in triggering DAS release than endogenous ROS.

### 2.4 In Vitro Photoactivatable Anti‐Angiogenic Ability of the Nanoparticles

**Figure 3:**
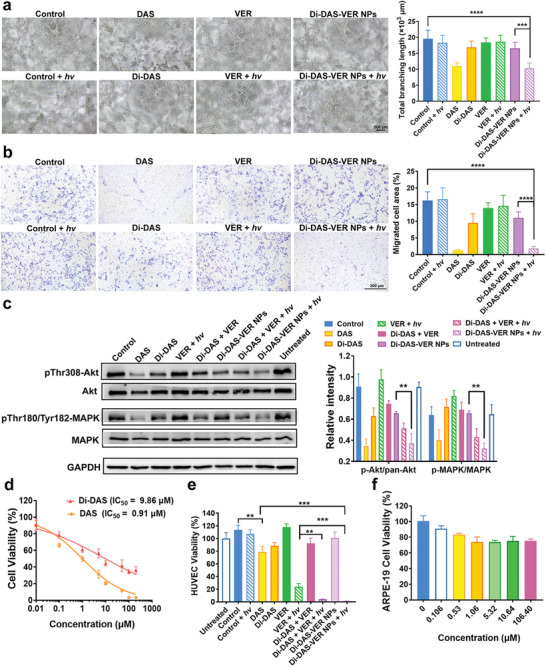
In vitro red light‐activatable anti‐angiogenic and endothelial cell killing abilities of Di‐DAS‐VER NPs. a) Representative images and the corresponding quantification results of VEGF‐stimulated HUVEC tube formation assay of Di‐DAS‐VER NPs at 4 h post‐treatment. HUVEC suspensions were treated with 20 ng mL^−1^ VEGF_165_ and co‐incubated with different formulations at a DAS equivalent dose of 0.05 µm, followed by 0.75 J cm^−2^ of light irradiation at 690 nm or not (10 mW cm^−2^, 75 s). The administrated dose of verteporfin was the same as that of Di‐DAS‐VER NPs determined by HPLC. *n* = 6. b) Representative images and the corresponding quantification results of HUVEC migration assay of Di‐DAS‐VER NPs at 24 h post‐treatment. HUVEC suspensions were seeded in the top chamber and incubated with different formulations at a DAS equivalent dose of 0.05 µm, followed by 0.75 J cm^−2^ of light irradiation at 690 nm or not. *n* = 6. c) Representative images and the corresponding quantification results of Western blot analysis of pro‐angiogenic signaling proteins. HUVECs were treated with different formulations at an equivalent dose of 1 µm DAS at 37 °C for 24 h, with or without 0.75 J cm^−2^ of 690 nm light irradiation. *n* = 3. d) Quantitative HUVEC viability of DAS or Di‐DAS treatment by MTT assay. Cells were incubated with 20 ng/mL VEGF_165_ and co‐treated with DAS or Di‐DAS at various concentrations for 48 h. *n* = 6. e) Quantitative HUVEC viability of Di‐DAS‐VER NP treatment by MTT assay. Cells were treated with 20 ng mL^−1^ VEGF_165_ and co‐incubated with different formulations at a DAS equivalent dose of 1 µm, followed by 4 J cm^−2^ of light irradiation at 690 nm or not (10 mW cm^−2^, 400 s). MTT assay was performed at 48 h after treatment. *n* = 6. f) Quantitative ARPE‐19 cell viability of Di‐DAS‐VER NP treatment by MTT assay. Cells were incubated with Di‐DAS‐VER NPs at various concentrations for 48 h. *n* = 6. Data were presented as mean ± standard deviations. **: *p* < 0.01; ***: *p* < 0.001; ****: *p* < 0.0001.

As aforementioned, DAS serves as an anti‐angiogenic agent by Src kinase blockade of VEGF‐mediated downstream signaling.^[^ ^28^ ^]^ Therefore, we hypothesized that 690 nm light irradiation could restore the anti‐angiogenic potential of the prodrug‐based nanoparticles by triggering prodrug cleavage and simultaneous DAS release. As a proof of concept, we systematically assessed the photoactivatable anti‐angiogenic ability of Di‐DAS‐VER NPs by tube formation assay in vitro. As shown in **Figure** **3**a and Figure S8 (Supporting Information), when incubated at a DAS equivalent dose of 0.1 µm, DAS totally inhibited endothelial reorganization into tube‐like networks, leading to a 43.8% decrease in the total branching length and a 62.5% decrease in the number of meshes compared with the control. In contrast, Di‐DAS hardly suppressed HUVEC tube formation, where the total branching length and the number of meshed were slightly reduced by 10.3 and 13.7%, respectively. The results confirm the significantly attenuated anti‐angiogenic ability of Di‐DAS due to the formation of prodrugs. Moreover, as expected, verteporfin plus irradiation, or Di‐DAS‐VER NPs without light irradiation could not block VEGF‐induced HUVEC differentiation and formation of vessel‐like tubes. Noteworthily, Di‐DAS‐VER NP treatment plus light irradiation resulted in significant inhibition of tube formation, with 47.4% and 60.0% reduction in the total branching length and the number of meshes, respectively, which was attributed to the fact that red‐light irradiation triggered the release of DAS against angiogenesis.

Encouraged by satisfactory results on light‐triggered inhibition of endothelial tube‐forming potential, we further explored the influence of Di‐DAS‐VER NPs on the migration of VEGF‐stimulated HUVECs by wound healing and transwell assays. Di‐DAS and Di‐DAS‐VER NP treatment without light exposure led to 67.6% and 49.8% of wound recovery, respectively. In contrast, Di‐DAS‐VER NPs plus light irradiation severely impaired the recovery of the generated scratches on HUVEC monolayer to 7.8%, comparable to that of DAS group (11.4%) (Figure S9, Supporting Information). Additionally, the cell number of HUVECs on the bottom side of the transwell, which represented the endothelial cells migrating across transwell membrane toward serum‐containing medium, was decreased by Di‐DAS‐VER NP incubation in the dark to some extent (32.2% relative to the control). However, Di‐DAS‐VER NPs plus light irradiation almost completely hindered HUVEC migration toward the lower compartment of the transwell, wherein the migrated cell area was significantly reduced by 88.6% relative to the control (Figure 3b). Generally, all these results suggest that the anti‐angiogenic ability of Di‐DAS‐VER NPs can be efficiently recovered after red‐light illumination.

Furthermore, we also checked the angiogenic signaling targets of HUVEC proliferation and survival by Western blot analysis. As presented in Figure 3c, Di‐DAS‐VER NP treatment plus light irradiation significantly inhibited the phosphorylation of Akt kinase, whose activity is essential for endothelial cell survival and migration,^[^ ^19^ ^]^ to nearly 0.41‐fold as compared to that of VEGF‐pretreated cells. Besides, Di‐DAS‐VER NP treatment had little impact on the phosphorylation of mitogen‐activated protein kinase (MAPK), a key signaling target in cell proliferation and vessel sprouting.^[^ ^19^ ^]^ Significant reduction in phosphorylation levels of MAPK was observed in Di‐DAS and VER plus light irradiation groups, ≈0.67‐fold relative to those of VEGF‐pretreated cells. As a comparison, the treatment of Di‐DAS‐VER NPs plus light irradiation led to the most remarkable downregulation of phosphorylated MAPK, about 0.50‐fold relative to that of VEGF‐pretreated cells. These data further corroborate the superior efficiency of Di‐DAS‐VER NPs for photoactivatable angiogenesis inhibition.

### 2.5 In Vitro Evaluation of Cytotoxicity and Biocompatibility of the Nanoparticles

We investigated whether the dimeric prodrug showed reduced cytotoxicity compared with DAS by 3‐(4, 5‐dimethylthiazol‐2‐yl) 2, 5‐diphenyl tetrazolium bromide (MTT) assay. As expected, Di‐DAS showed significantly decreased cytotoxicity, evidenced by the 50% inhibition concentration (IC_50_) for Di‐DAS at 9.86 µm, ≈ten‐fold higher than that of DAS (0.91 µm) (Figure 3d). The results imply that undesired systemic anti‐angiogenic effects could be minimized due to inactivity of Di‐DAS.

In view of the primary roles of neovascular endothelium in CNV progression, MTT assay was then executed to measure the VEGF‐stimulated HUVEC viability after treatments with different formulations. As a comparison to DAS treatment and VER treatment followed by light irradiation, Di‐DAS‐VER NPs exhibited significantly enhanced cytotoxicity when exposed to 10 mW cm^−2^, 400 s of light irradiation at 690 nm (Figure 3e). The superior cell‐killing effects could be credited to photoactivated PDT and anti‐angiogenic ability of the multifunctional nanosystem. In this regard, we performed the synergy analysis of DAS and VER plus light irradiation. We observed synergy scores of DAS and PDT combinations ranged between 6.76 and 36.17 within the equivalent DAS concentrations of 0.1–10 µm, with the mean score of 15.65. These results demonstrated the likely synergistic cytotoxicity effects of DAS and PDT (Figure S10, Supporting Information).

We also examined the in vitro biocompatibility of the nanoparticles in HUVECs and human retinal pigment epithelial cells (ARPE‐19). As shown in Figure S11 (Supporting Information) and Figure 3f, after exposure to a high dose of Di‐DAS‐VER NPs (106.4 µm) in the dark for 48 h, both cell lines still showed high cell viability of ≈80%, suggesting that the nanoparticles have little cytotoxicity to normal vascular endothelium and retinal pigment epithelium without light irradiation.

### 2.6 In Vivo Pharmacokinetic Performance of Di‐DAS‐VER NPs and Red Light‐Triggered Intraocular DAS Release

**Figure 4:**
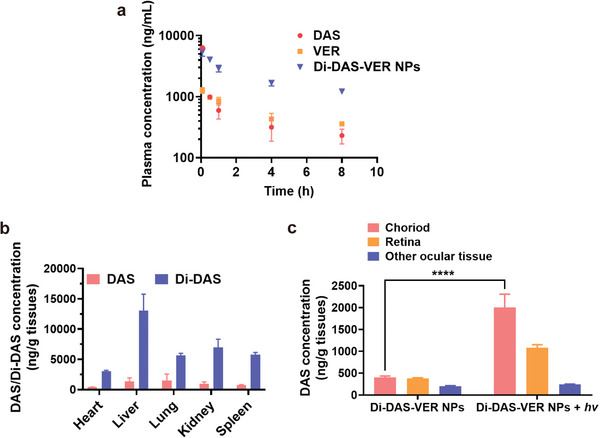
Pharmacokinetic profiles and in vivo biodistribution of Di‐DAS‐VER NPs. a) Plasma concentration‐time curve of the mice following intravenous injection of different formulations at the equivalent dose of 42 mg kg^−1^ DAS. The administrated dose of VER was the same as that of Di‐DAS‐VER NPs determined by HPLC. b) HPLC quantitative analysis of DAS/Di‐DAS in the main organs at 2 h post‐intravenous administration of Di‐DAS‐VER NPs in CNV mice. c) LC‐MS/MS quantitative analysis of intraocular DAS distribution in CNV mice at 2 h post‐intravenous injection of Di‐DAS‐VER NPs. The irradiated eyes were exposed to 25 J cm^−2^ of 690 nm laser illumination at 1 h post‐injection. Data are presented as mean ± standard deviations (*n* = 3). ^****^ *p* < 0.0001.

Prior to evaluating the in vivo therapeutic efficacy of Di‐DAS‐VER NPs, we first examined the pharmacokinetic behaviors of Di‐DAS‐VER NPs in C57BL/6J mice by HPLC analysis of plasma samples. As shown in **Figure** **4**a, there was a fast decrease in plasma drug concentration of DAS for the mice receiving free drug solution within 4 h, to 4.97% relative to the plasma drug concentration at 5 min. Interestingly, systemic plasma clearance of Di‐DAS‐VER NPs in the mice was notably slower. There was still 30.78% of Di‐DAS‐VER NPs retained in the circulation at 4 h post‐injection. The estimated plasma half‐life of Di‐DAS‐VER NPs was ≈6.1 h in terms of Di‐DAS content, 2.17‐times and 1.22‐times higher than free DAS and VER, respectively (Table S1, Supporting Information). The extended systemic circulation of Di‐DAS‐VER NPs was consistent with previous reports that amphiphilic PEGylated nanoparticles helped overcome the limitations of free drug form, including poor stability and short half‐life.^[^ ^29^, ^30^ ^]^

Next, we studied the biodistribution of Di‐DAS‐VER NPs in laser‐induced CNV mice, a well‐established rodent model for wAMD research.^[^ ^31^ ^]^ To validate our hypothesis that red‐light irradiation to the eye could trigger intraocular DAS release, CNV mice were intravenously administrated with Di‐DAS‐VER NPs, followed by 690 nm laser irradiation (25 J cm^−2^, half of the clinical‐standard irradiation dose^[^ ^32^ ^]^) to the mouse eyes at 1 h post‐injection or not. Then the mice were euthanized at 2 h post‐injection. Their main organs and eyeballs were harvested for analysis of drug content. As shown in Figure 4b, the DAS concentrations remained at low levels in the main organs without light exposure (433.6–1399.3 ng g^−1^ tissue) after Di‐DAS‐VER NP administration. The DAS concentrations were less than 20% relative to those of DAS‐treated mice (Figure 4b; Figure S8, Supporting Information). More importantly, there was a remarkable elevation of DAS concentration in the choroidal tissues of the irradiated eyes of Di‐DAS‐VER NPs‐treated mice (2005.89 ng g^−1^), which was ≈5.02‐times higher than the choroid of the unirradiated eyes (399.84 ng g^−1^ tissue) (Figure 4c). As a comparison, for the mice administrated with free DAS plus VER solutions, the majority of DAS was detected in the main organs (Figure S12, Supporting Information). Such unselective distribution of DAS posed potential toxicity to healthy organs. Taken together, these findings provide direct evidence that Di‐DAS‐VER NPs specifically release anti‐angiogenic DAS in the ocular tissues upon red‐light irradiation, especially in the diseased choroid, with minimized systemic exposure of DAS after intravenous administration.

### 2.7 Suppression of Experimental CNV by Di‐DAS‐VER NPs In Vivo.

**Figure 5:**
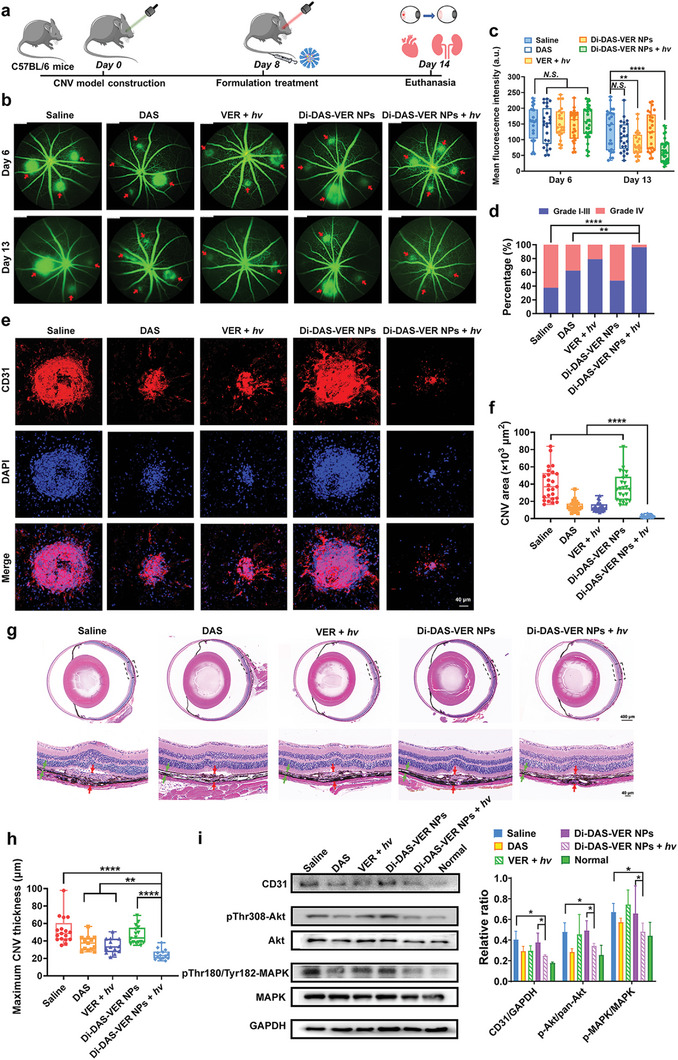
CNV regression by Di‐DAS‐VER NPs in the laser‑induced CNV mouse model. a) Schematic illustration of the treatment process with different formulations with or without light irradiation. Laser‐induced CNV mice were intravenously injected with different formulations at an equivalent dose of 6 mg m^−2^ of VER on day 8, with or without 25 J cm^−2^ of 690 nm light irradiation at 1 h post‐injection. b,c) Representative fundus fluorescein angiography images and the corresponding quantification results of mean fluorescence intensity on days 6 and 13. Red arrows denote the leaking CNV regions. *n* = 8 mice, 24–28 lesions. d) Clinical grading of the fluorescein leakage degree on day 13 according to (b,c). Grade I: No hyperfluorescent leakage; Grade II: Hyperfluorescence without leakage; Grade III: Hyperfluorescent leakage; Grade IV: Significantly pathological leakage. *n* = 8 mice, 24–28 lesions. e,f) Representative confocal images of anti‐CD31 immunofluorescence‐stained vessels of RPE‐choroid flat mounts and the corresponding quantification results of the CNV areas. *n* = 6 mice, 24 lesions. g,h) Representative images of H&E staining of paraffin‐embedded eye sections and the corresponding quantification data of CNV lesion thickness. The dashed boxs denote the magnified view of the CNV lesions shown in the lower images. Red arrows denote the maximum thickness of choroidal lesions and green arrows denote the adjacent normal choroid. *n* = 6 mice, 13–17 lesions. i) Representative images and the corresponding quantification results of Western blot analysis of pro‐angiogenic signaling proteins in RPE‐choroid samples of CNV mice treated with different formulations. *n* = 3. Data are presented as mean ± standard deviations. *N.S*.: No statistical significance; ^*^ *p* < 0.05, ^**^ *p* < 0.01, and ^****^ *p* < 0.0001.

We subsequently investigated the beneficial effects of Di‐DAS‐VER NPs in laser‐induced CNV mice (**Figure** **5**a), for testing whether the anti‐angiogenic and PDT combination strategy could effectively regress CNV. First, we used fundus fluorescein angiography (FFA) to visualize the vascular leakage of CNV lesions on days 6 and 13, before and after the indicated treatments. As shown in Figure 5b,c, there was severe hyperfluorescent neovascular leakage 6 days after laser photocoagulation, verifying the successful establishment of CNV model. Those CNV mice subject to saline or Di‐DAS‐VER NPs treatment without light irradiation still showed strong vascular leakage on day 13 (5 days after the treatments), with the average leakage intensity at 87.1% and 87.8% relative to day 6. Notably, Di‐DAS‐VER NPs plus light irradiation achieved a substantial reduction in neovascular leakage, evidenced by the average leakage intensity declining to 38.6% relative to day 6. While monotherapy groups with DAS or VER plus light irradiation showed insufficient ablation of neovascular leakage to 76.1% and 55.9% relative to day 6, respectively. Furthermore, clinical grading of CNV leakage showed that as high as 62.5% of lesions of the saline group were recognized as grade IV (significantly pathological leakage) (Figure 5d). In contrast, the predominant proportion (96.4%) of lesions of the Di‐DAS‐VER NPs plus light irradiation group fell in grade I‐III (no hyperfluorescent leakage, hyperfluorescence without leakage or hyperfluorescent leakage), statistically significantly altered from the saline group. In addition, 37.5% and 20.8% of lesions of DAS and VER plus light irradiation groups, respectively, were still defined as grade IV. Based on these results, we could conclude that the most significant inhibition of CNV leakage was achieved by Di‐DAS‐VER NPs plus light irradiation.

Further, immunofluorescence staining of CD31, an extensively used vascular endothelium marker,^[^ ^31^ ^]^ was utilized to stain the retinal pigment epithelium (RPE)‐choroid flat mounts for quantification analysis of the CNV area. As shown in Figure 5e,f, Di‐DAS‐VER NPs plus light irradiation treatment almost achieved complete CNV occlusion. The average CNV area of those mice declined to 2.47 × 10^3^ µm^2^, 6.19% relative to that of the saline‐treated mice (39.98 × 10^3^ µm^2^). Moreover, the CNV area of the Di‐DAS‐VER NPs plus light irradiation group was evidently less than other groups, including the mice receiving monotherapies (14.51 × 10^3^ µm^2^ and 13.59 × 10^3^ µm^2^ for DAS and VER plus light irradiation groups, respectively) or Di‐DAS‐VER NPs without light exposure (36.67 × 10^3^ µm^2^). In consistence, the most significant reduction in the vertical dimensions of CNV was observed in the haematoxylin and eosin (H&E) stained eyeball cross‐sections of the Di‐DAS‐VER NPs plus light irradiation group (Figure 5g,h). The mean maximum CNV thickness was decreased to 24.43 µm after Di‐DAS‐VER NPs plus light irradiation treatment. In contrast, this pathological indicator remained as high as 52.24 µm, 37.38 µm, and 34.27 µm for those mice administered with saline, DAS, and VER plus light irradiation, respectively. Additionally, Western blot data also revealed that Di‐DAS‐VER NPs plus light irradiation treatment resulted in a 39.6% reduction of CD31 expression in RPE‐choroid samples. Also, there was a decline of phosphorylation levels of angiogenic signaling proteins Akt and MAPK in the Di‐DAS‐VER NPs plus light irradiation group, to ≈70% relative to the saline‐treated mice (Figure 5i).

### 2.8 Biosafety Evaluation

**Figure 6:**
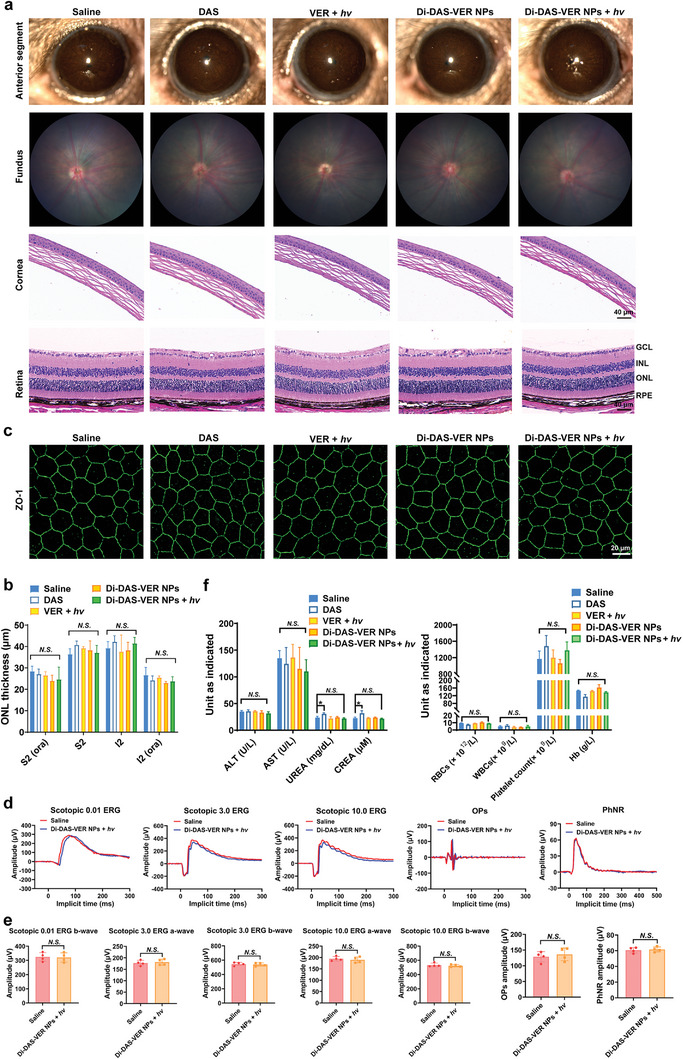
In vivo biosafety evaluation of different formulation treatments in the laser‑induced CNV mouse model. a) Representative slit‐lamp images of the anterior segment, fundus photography, and photomicrographs of H&E‐stained cornea/retina slices of the mice after different formulation treatments. b) Quantitative analysis of retinal ONL thickness of H&E‐stained eye cross‐sections of the mice after different formulation treatments. *n* = 4. c) Representative confocal images of anti‐ZO‐1 immunofluorescence‐stained tight junctions of RPE from RPE‐choroid flat mounts. d) Representative wave responses of scotopic electroretinogram (ERG) signals (0.01, 3.0, and 10.0 cd s m^−2^), oscillatory potentials (OPs), and photopic negative response (PhNR) of the mice treated with saline or Di‐DAS‐VER NPs plus light irradiation. e) Quantification analysis of the amplitudes of scotopic a‐wave and b‐wave, OPs, and PhNR corresponding to (d). *n* = 4. f) Hematological indicators and biochemical blood profiles of the mice after different formulation treatments. Mice were intravenously administrated with different formulations at an equivalent dose of 6 mg m^−2^ of VER, with or without 25 J cm^−2^ of 690 nm light irradiation at 1 h post‐injection. RPE, retinal pigment epithelium; ONL, outer nuclear layer; INL, inner nuclear cell layer; GCL, ganglion cell layer; S2, superior quadrant; I2. inferior quadrant; ora, vicinity of ora serrata; ALT, alanine aminotransferase; AST, aspartate aminotransferase; CREA, creatinine. Data are presented as mean ± standard deviations. *n* = 4. *N.S*.: No statistical significance; ^*^ *p* < 0.05.

PDT with VER (690 nm, 50 J cm^−2^) has already become one of the clinical treatment modalities for wAMD.^[^ ^32^ ^]^ We also confirmed that the utilized light irradiation procedure (25 J cm^−2^, 80 mW cm^−2^ for 312 s) caused negligible temperature elevation to the mouse eyes, indicating that the 690 nm laser application would not cause thermal damage to the eyes (Figure S13, Supporting Information). Despite this, we carefully evaluated the ocular toxicity after the in vivo studies, especially for those treatments that involved the 690 nm laser application to the mouse eyes. Mainly, there were no signs of clouding, ulceration, or hemorrhage either in the anterior chamber or fundus according to slit‐lamp images and fundus photographs of the mice treated with Di‐DAS‐VER NPs plus light irradiation (**Figure** **6**a). Besides, we confirmed similarities of H&E images of corneal and retinal sections among the saline‐treated mice and those receiving different formulation treatments (Figure 6a). The quantification data of retinal outer nuclear layer (ONL) thickness of the mice treated with Di‐DAS‐VER NPs plus light irradiation did not differ from the non‐irradiated eyes (Figure 6b).

To evaluate in vivo biocompatibility of Di‐DAS‐VER NPs on RPE, we performed the immunofluorescence staining of zonula occludens‐1 (ZO‐1) on RPE‐choroid flat mounts, which is the main structural protein of RPE intercellular junctions.^[^ ^33^ ^]^ As revealed in Figure 6c, RPE‐choro flat mounts of the mice treated with Di‐DAS‐VER NPs plus light irradiation exhibited densely packed ZO‐1‐stained hexagonal cellular boundaries, similar to the control. The organized ZO‐1 expression demonstrated no obvious toxicity of Di‐DAS‐VER NPs on RPE tight junctions. Moreover, we also confirmed that the treatments did not impair the visual function through electroretinogram (ERG) assessments, given that there was no distinguishable alteration of ERG signals on the mice receiving Di‐DAS‐VER NPs plus light irradiation treatment compared to the control (Figure 6d,e). Overall, these results indicate the excellent ocular biosafety of the proposed treatment.

Finally, to further demonstrate the biosafety of the intravenous nanosystem, hematological parameters of the liver and kidney functions, including aspartate aminotransferase (AST), alanine aminotransferase (ALT), urea, and creatinine (CREA), were characterized after the mice were administered with DAS or Di‐DAS‐VER NPs. To our notice, the DAS‐treated mice exhibited hematuria within 24 h post‐administration. In addition, there was ≈0.2‐fold elevation in serum urea and creatinine levels of the DAS‐treated mice (Figure 6f), which implies potential kidney dysfunction after intravenous administration with free DAS. In contrast, all measured biochemical indicators of the Di‐DAS‐VER NPs group were closely similar to those of the control group. The results reveal that Di‐DAS‐VER NPs displayed significantly reduced toxicity compared with free DAS. Meanwhile, no histopathological changes could be found in H&E‐stained main organ sections of the mice after Di‐DAS‐VER NP treatment (Figure S14, Supporting Information). Taken together, these data suggest favorable biosafety of the prodrug‐based nanosystem.

## 3 Discussion and Conclusion

The unique transparent cavity of the eye makes photoresponsive systems ideal tools to achieve drug delivery to the posterior of eyes safely and efficiently. Hence, it is inspiring to exploit photoresponsive platforms as noninvasive therapeutics for retinal and choroidal diseases, which currently require monthly intravitreal administration for disease management. Nowadays, there are growing explorations in photoresponsive nanocarriers for targeted drug delivery, sustained payload release in the eye, or minimally invasive phototherapy of diseased lesions, including nanoparticles, intravitreal drug depots, intraocular lenses, hydrogels, etc.^[^ ^22^, ^34^, ^35^, ^36^, ^37^, ^38^, ^39^, ^40^ ^]^ However, most reported studies used UV or blue light to trigger delivery systems, which can be harmful to the eye, especially the retina.^[^ ^41^ ^]^ In our previous work, we have demonstrated that green‐light irradiation (505 nm) could trigger the disassembly of intravenously‐administered photoresponsive nanocarriers to release hydrophobic drugs, which can extravasate into tumor tissues by crossing the inner blood‐retinal barriers more efficiently than nanoparticles.^[^ ^38^ ^]^ The study validated the photo‐enhanced drug accumulation as a promising option for systemic treatment of eye diseases. Considering that long‐wavelength light (e.g., red or near‐infrared (NIR) light) could be an appropriate irradiation source due to its deep tissue penetration and reduced risks of phototoxicity to the eye,^[^ ^41^, ^42^, ^43^ ^]^ we have been further working on the development of ophthalmic nanosystems that can respond to red or NIR light. Conventional red/NIR light‐responsive systems mainly rely on the two‐photon excitation mechanism or photon upconversion process, where lasers equipped with high‐power sources are required.^[^ ^42^, ^44^ ^]^ Besides, these formulations often require complex fabrication processes or contain rare‐earth elements in the upconversion nanoparticles, which pose challenges for clinical use.^[^ ^45^ ^]^

To resolve the safety concerns and enhance the therapeutic efficacy against wAMD, we strategically exploited the photodynamic process to mediate red light‐triggered activation of nanosystems through singlet oxygen‐induced photocleavage. To our knowledge, this cleavage strategy has not been applied for systemic delivery of an anti‐angiogenic agent and a photosensitizer to the posterior of the eye as combination therapeutics. The prodrug‐based nanosystem presented here was designed based on the FDA‐approved agents and biocompatible polymer. Besides, our proposed nanosystem only involves simple chemical synthesis and one‐step nanoprecipitation method for preparation, which allows for the potential of scale‐up production. Since the nanoparticles can be long‐circulated in the blood after intravenous injection, the highly vascularized structure of choroid can facilitate nanoparticle perfusion, especially for the CNV lesions.^[^ ^46^, ^47^ ^]^ As proof of concept, intravenous administration of such prodrug‐based nanoparticles showed minimized systemic side effects, due to the compromised potency of the prodrug without activation by light. Meanwhile, red‐light irradiation to mouse eyes triggers the PDT effect and recovers the function of anti‐angiogenic agents from the circulating nanoparticles to destroy the nearby CNV vessels. Due to the superior efficacy of combination treatment, a low‐dose laser fluence (25 J cm^−2^, half of the clinical‐standard irradiation dose) can achieve desirable CNV closure. The utilized parameters have been reported safe^[^ ^48^, ^49^ ^]^ and proved little damage to the overlying retina in our experiments.

In summary, we exploited a red light‐triggered intraocular drug release strategy to achieve anti‐angiogenic and PDT combination treatment of wAMD via less‐invasive intravenous administration. As proof of concept, red light‐activatable nanoparticles (Di‐DAS‐VER NPs) were successfully fabricated with anti‐angiogenic prodrug Di‐DAS, photosensitizer verteporfin, and biocompatible lipid. Di‐DAS‐VER NPs can be intravenously injected into a standard CNV mouse model, and activated by red‐light irradiation (690 nm) on the mouse eyes. We have demonstrated that the light irradiation enabled PDT‐induced vascular occlusion combined with intraocular DAS release for anti‐angiogenesis, which greatly strengthened CNV suppression effect compared with monotherapies. Moreover, Di‐DAS‐VER NPs exhibit excellent biocompatibility and inactivity in normal tissues without light exposure. There was no sign of systemic or ocular toxicity after Di‐DAS‐VER NP treatment. Our study may provide a promising clinical solution for minimally‐invasive administration of combination therapeutic agents for angiogenic eye diseases.

## 4 Experimental Section

### Materials

Dasatinib (DAS), N, N’‐Dicyclohexylcarbodiimide (DCC) and N, N‐dimethylaminop‐yridine (DMAP), and verteporfin were purchased from Macklin Biochemical Co., Ltd (Shanghai, China). DSPE‐PEG_2000_ was purchased from Ponsure Biotechnology Co., Ltd. (Shanghai, China). 3,3′‐(propane‐2,2‐diylbis(sulfanediyl)) dipropionic acid was obtained from Ruixi Biotechnology Co., Ltd. (Xi'an, China). 3‐(4, 5‐dimethylthiazol‐2‐yl) 2, 5‐diphenyl tetrazolium bromide (MTT) and 2′‐7′dichlorofluorescin diacetate (DCFH‐DA) were purchased from Sigma‐Aldrich Inc (St. Louis, MO, USA). Singlet Oxygen Sensor Green (SOSG) and Pierce BCA Protein Assay Kit were purchased from Thermo Fisher Scientific, Inc. (Eugene, OR, USA). The human umbilical vein endothelial cells (HUVEC) and human retinal pigment epithelial cells (ARPE‐19) were obtained from American Type Culture Collection (Manassas, VA, USA). Gibco Dulbecco's Modified Eagle medium (DMEM), fetal bovine serum (FBS), penicillin‐streptomycin solution and trypsin‐EDTA 0.25% solution, collagen I (rat tail) were purchased from Thermo Fisher Scientific, Inc. (Eugene, OR, USA). Standard Matrigel extracellular matrix was obtained from Corning, Inc. (New York, NY, USA). All chemicals used were analytical or ACS reagent grade.

### Instruments

Dynamic light scattering (DLS) analysis was conducted by Nano‐ZS (Malvern Instruments, UK). Transmission electron microscopy (TEM) images were captured by CM100 Transmission Electron Microscope (Philips, USA). UV‐Vis absorption spectra and cell viability were measured by SpectraMax M4 multi‐mode microplate reader (Molecular Devices, USA). Drug‐loading capacity, encapsulation efficiency, and drug concentrations were measured by 1260 Infinity II HPLC (Agilent Technologies, USA). Photolysis experiments and irradiation were conducted using a commercial diode laser system (Laserwave, Canada). The light irradiance was measured by PM100USB power and energy meter (Thorlabs, USA) equipped with S142C integrating sphere photodiode power sensor (Si, 350–1100 nm, Thorlabs, USA). Fluorescence images were captured by LSM 980 confocal microscope (ZEISS, German). Intraocular DAS content was detected by API 4000 liquid chromatograph‐triple quadrupole mass spectrometry (AB Sciex, USA). Experimental CNV mouse model was constructed using OcuLight Infrared Laser System (IRIDEX, USA). Anterior chamber and fundus images were acquired by SL‐D701/BG‐5/IMAGEnet digital slit lamp photography system (Topcon, Japan) and Micron IV retina imaging system (Phoenix Research Laboratories, USA), respectively. Electroretinography (ERG) tests were examined by Celeris rodent ERG system (Diagnosys LLC, USA).

### Animals

C57BL/6J mice (18–22 g, 6–8 w, male) were obtained from the Animal Laboratory of Zhongshan Ophthalmic Center. All the mice were housed in conventional experimental holding areas with an alternating 12 h light/dark‐period control, regulated temperature at 21 ± 4 °C, relative humidity at 50 ± 10% and ad libitum feeding to UV‐treated food and 1‐µm‐filtered water. The animal studies were carried out under the approval of the Institutional Animal Care and Use Committee (IACUC), Zhongshan Ophthalmic Center, Sun Yat‐sen University (IACUC No. Z2022059).

### Synthesis of ROS‐Cleavable Dimeric Prodrug

The dimeric prodrug Di‐DAS was synthesized through an esterification reaction with ROS‐sensitive thioketal‐containing dipropionic acid. Briefly, dasatinib, 3,3′‐(propane‐2,2‐diylbis(sulfanediyl)) dipropionic acid, DCC, and DMAP were mixed in a reaction flask, followed by evacuation and nitrogen aeration three times. Then the reactants were fully dissolved in anhydrous DMF and stirred for 48 h at room temperature. The product was further purified by a silica gel column chromatography system. Mass spectroscopy and proton nuclear magnate renascence (^1^H NMR) spectra were obtained for the characterization of the resulting product.

### Nanoparticle Preparation

The prodrug‐based nanoparticles were prepared by the nanoprecipitation method as previously described.^[^ ^42^ ^]^ In brief, DSPE‐PEG_2000_, verteporfin, and Di‐DAS were co‐dissolved in DMSO at optimized feeding ratios of 1/15 (verteporfin: Di‐DAS, wt./wt.) and 1/5 (PEG‐lipid: Di‐DAS, wt./wt.), and then dripped dropwise into water under stirring to facilitate the self‐assembly of prodrug‐incorporated nanoparticles. The nanoparticle solution was centrifuged at 2 × 10^4^ rpm and 4 °C for 15 min to remove the incorporated components. Subsequently, the nanoparticle precipitate was re‐suspended in distilled water.

### Nanoparticle Characterization

The hydrodynamic diameter, polydispersity index (PDI), and surface charge of the nanoparticles were measured by DLS analysis. TEM imaging was applied to observe the morphology of the nanoparticles. HPLC analysis was conducted to examine encapsulation efficiency (EE%) and drug‐loading capacity (DLC%) of the nanoparticles.

### Colloidal Stability Evaluation

The stability of the nanoparticles was evaluated by DLS analysis and TEM imaging after incubation with PBS buffer (pH 7.4) and serum‐containing DMEM medium (10%) at 37 °C for at least 48 h. The size changes of nanoparticles can indicate their colloidal stability.

### Singlet Oxygen (^1^O_2_) Detection

Free verteporfin and nanoparticle solution were mixed with SOSG to make final concentrations of verteporfin at 2 mm and SOSG at 10 µm in water. Next, the solution was irradiated by a commercial diode laser at 100 mW cm^−2^ for various periods (0, 10 s, 20 s, 30 s, 1 min, 2 min). SOSG solution without photosensitizer also received the light irradiation as the control group. The SOSG fluorescence intensity was then measured by a multi‐mode microplate reader (Ex = 504 nm, Em = 525 nm).

### Red Light‐Triggered Drug Release

After 690 nm laser irradiation at 100 mW cm^−2^ for various intervals (0, 1, 2, 5, 10 min), the photocleavage products released from the nanoparticle solutions (5 µm of Di‐DAS) were separated by centrifugation, fully dissolved in acetonitrile/methanol mixture (1:1) and further analyzed by HPLC. The cumulative drug release in the dark was evaluated as the control for drug leakage calibration before illumination.

Besides, DAS release from Di‐DAS‐VER NPs upon normal or pathological ROS levels was evaluated with dialysis method under sink conditions. Briefly, 10 µL of Di‐DAS‐VER NPs were added into dialysis bags (MWCO 100 000 Da), fully immersed in 7 mL of the release media containing 1 µm H_2_O_2_ or 1 mm H_2_O_2_, and incubated at 37 °C in a shaking water bath (120 rpm). At the indicated time points, the release media were replaced with the same volume of fresh media and then analyzed by HPLC. Di‐DAS degradation rate (%) was calculated as the percentage of decomposed Di‐DAS versus initial Di‐DAS content (*t* = 0). DAS recovery percentage (%) was identified as the molar ratio of generated DAS versus the doubled amount of initial Di‐DAS content.

### Intracellular ROS Detection

HUVECs were seeded on confocal dishes (2.0 × 10^5^ cells per dish) overnight, followed by the incubation with various formulations (0.1 µm verteporfin) for an additional 4 h. And then the cells were washed with PBS buffer three times and incubated with 10 µm of DCFH‐DA at 37 °C for 20 min. After that, the cells were gently washed with PBS buffer to remove extracellular DCFH‐DA probes, and then exposed to 690 nm laser irradiation (10 mW cm^−2^, 400 s). The fluorescence signal of intracellular ROS was observed under the confocal microscope (Ex = 488 nm, Em = 525 nm).

### In Vitro Angiogenesis Assays

A variety of angiogenesis assays have been widely used to assess the angiogenic potential of endothelial cells. First, tube formation assay was employed to assess the anti‐angiogenic capacity of the prodrug‐based nanosystem to inhibit VEGF‐stimulated endothelial tube growth. In brief, 0.1 mL of HUVEC suspension (1.5 × 10^4^ cells per well) in basal medium was gently mixed with different formulations (PBS, DAS, Di‐DAS, verteporfin, and Di‐DAS‐VER NPs; at the DAS equivalent concentration of 0.1 µm), and then seeded in Matrigel‐coated 96‐well plates. For the irradiation groups, 690 nm laser light (10 mW cm^−2^, 75 s) was applied directly on cell culture with a diode laser collimating system. The images were captured at 4 h to observe the differentiation and formation of vessel‐like tubes. The total branching length and the number of meshes of tube formation were analyzed by the angiogenesis analyzer toolset of ImageJ.

The effect of the nanosystem to attenuate the migration of VEGF‐induced HUVECs was evaluated by wound‐healing assay and transwell assay. Briefly, for wound‐healing assay, HUVECs were cultured in a collagen‐coated 24‐well plate (4 × 10^5^ cells per well). When cell confluency reached ≈90%, scratches were created with a sterile 200 µL‐pipette in the middle of the HUVEC monolayer. Then the cells were washed by PBS buffer for three time, and subsequently received different formulation treatments as aforementioned. To quantify the scratch widths, images were recorded at 0 h and 20 h after the treatments and analyzed by ImageJ. The recovery rates could be calculated as (wound closure area/original scratch area) × 100%.

For transwell assay, 0.1 mL of HUVEC suspended in basal medium were seeded in the upper compartment of 24‐well transwell plates (2.5 × 10^5^ cells per well). Meanwhile, 0.65 mL of culture medium with 10% FBS was added into the basement. The cells were treated with various formulations as described above. After 24 h of incubation, the cells located on the upper side of transwell membrane were gently removed by wiping with cotton swabs. Subsequently, the cells that actively migrated downward and attached to the bottom of the lower compartment were detected by 1% crystal violet staining. The migrated cell area per field was quantified by the particle analysis function of ImageJ.

### In Vitro Cytotoxicity and Biocompatibility Assessment

For cytotoxicity evaluation, HUVECs were seeded on 96‐well plates (5.0 × 10^3^ cells per well) and cultured overnight. Then the cells were incubated with a range of concentrations of DAS or Di‐DAS (0–200 µm). In another experiment, the cells were treated with various formulations (DAS, Di‐DAS, verteporfin, and Di‐DAS‐VER NPs; at the DAS equivalent concentration of 2 µm) for 8 h, followed by being washed with PBS buffer three times and then irradiated (10 mW cm^−2^, 400 s) or not. For synergy analysis of DAS and PDT, HUVECs were treated with 20 ng mL^−1^ VEGF_165_ and co‐incubated with DAS and VER at the DAS equivalent concentrations of 0.1–10 µm, followed by 690 nm light irradiated (10 mW cm^−2^, 400 s). After incubation for 24 h or 48 h, 10 µL of MTT solution (5 mg mL^−1^) was added into each well and incubated for 4 h. Afterwards, the culture medium was replaced with 200 µL of DMSO to fully dissolve the generated formazan crystals. With the UV–Vis absorbance at 570 nm detected by a multi‐mode microplate reader, cell viability was calculated as the percentage of control. Synergy scores were calculated by SynergyFinder Plus with Zero interaction potency (ZIP) mode.^[^ ^50^ ^]^

To evaluate the in vitro biocompatibility of the prodrug‐based nanosystem, HUVECs and ARPE‐19 cells were treated with nanoparticle solution at various concentrations in the dark for 48 h (DAS equivalent concentration of 0–106.4 µm), followed by the measurement of cell viability by MTT assay as described above. The cells without treatment were prepared as the control.

### Construction of Laser‐Induced CNV Mouse Model

The experimental CNV mouse model that closely simulates wet AMD pathology was established according to the previous report.^[^ ^22^, ^31^ ^]^ Briefly, C57BL/6 mice (6–8 w) were anesthetized with 1% pentobarbital sodium (50 mg kg^−1^). Then their pupils were instilled with 0.5% tropicamide eye drops and tetracaine eye drops, followed by carboxymethylcell‐hypromellose topical drops for pupil dilation, topical anesthesia, and hydration, respectively. Each mouse's eyes were applied with four laser burns (810 nm wavelength, 120 mW of power, 75 µm of spot size, 75 ms of duration) with OcuLight Infrared Laser System at almost equal distance to the optic nerve head. The production of vaporization bubbles upon laser photocoagulation indicated the rupture of the Bruch's membrane and successful induction of CNV lesions.

On day 8, CNV model mice were administrated with a single intravenous injection of different formulations at the equivalent dose of 6 mg m^−2^ of verteporfin. Then the mice were anesthetized by intraperitoneal administration of 1% pentobarbital sodium (50 mg kg^−1^). Their pupils were thereafter dilated with 0.5% tropicamide eye drops, followed by topical anesthesia with tetracaine eye drops. Before the light irradiation application, mouse corneas were lubricated with carboxymethylcell‐hypromellose topical drops. At 1 h post‐intravenous injection, the mouse eyes were irradiated with 690 nm laser (25 J cm^−2^, 80 mW cm^−2^ for 312 s), which was set to be half of the clinically used power energy. Then mice were placed on a heating pad in the recovery cages after the treatment. Besides, healthy mice and non‐treated CNV mice were used as the control. All animal procedures were carried out following the Institutional Animal Care and Use Committee (IACUC), Zhongshan Ophthalmic Center, Sun Yat‐sen University.

### Pharmacokinetics and Biodistribution Studies

For the characterization of pharmacokinetic behavior of Di‐DAS‐VER NPs, mice were intravenously administrated with different formulation solutions at the equivalent dose of 42 mg kg^‐1^ DAS. Then blood samples (about 25 µL) were harvested in the heparinized tubes via retro‐orbital sampling at the predetermined time points (5 min, 30 min, 1 h, 4 h, and 8 h), followed by centrifugation at 1000 g for 10 min at 4 °C for plasma preparation. Subsequently, 10 µL of plasma samples were mixed with 40 µL of the acetonitrile‐methanol mixture (1:1) for protein precipitation. After ultra‐centrifugation at 2 × 10^4^ g for 15 min at 4 °C, the supernatant was collected for HPLC analysis of free drugs and the prodrug. The pharmacokinetic parameters were analyzed by PKSolver program with noncompartmental data analysis mode.

To investigate whether the photoresponsive nanoparticles can achieve intraocular drug accumulation via systemic administration, laser‐induced CNV mice were intravenously administrated with free drugs or Di‐DAS‐VER NPs at the equivalent concentration of 42 mg kg^−1^ DAS, followed by 690 nm light irradiation or not at 1 h post‐injection (25 J cm^−2^). After that, the mice were immediately sacrificed by CO_2_ asphyxiation and followed by cervical dislocation at 2 h post‐injection. Then the eyes and main organs (heart, lung, kidney, spleen, and liver) of the mice were surgically excised for preparation of tissue homogenate samples. The free drugs and prodrug in the main organ homogenate were measured by HPLC. Besides, the DAS concentration of ocular samples was detected by LC‐MS/MS analysis. MS/MS parameters for DAS detection were set as follows: positive ionization mode; precursor/product ion pairs (m/z) of 488.2/401.1; 10 V of collision energy; 60 V of delustering potential; 15 V of collision cell exit potential.

### Fundus Fluorescein Angiography (FFA)

Fundus fluorescein angiography analysis was performed to examine the CNV leakage on days 6 and 13. Briefly, the mice were intraperitoneally injected with 0.2 mL of 2% fluorescein sodium after anesthesia and pupil dilation. Then FFA images were taken at ≈5 min post‐fluorescein injection by Micron V imaging system. Clinical grading of CNV leakage degrees was performed by two masked researchers following the reported criteria:^[^ ^51^ ^]^ Grade I: No hyperfluorescent leakage; Grade II: Hyperfluorescence without leakage; Grade III: Hyperfluorescent leakage; Grade IV: Significantly pathological leakage.

### Electroretinogram (ERG) Recording

After treatment with saline or Di‐DAS‐VER NPs plus light irradiation as aforementioned, the mice were dark‐adapted overnight. Before the ERG test, the mice were anesthetized and administered with 0.5% tropicamide eye drops and tetracaine eye drops for pupil dilation and topical anesthesia. Then the electrodes were placed on the corneas after carboxymethylcell‐hypromellose topical drop instillation. Scotopic ERG under three types of light intensity (0.01, 3.0 and 10.0 cd s m^−2^) and light‐adapted flash ERG‐photopic negative response (PhNR) after 10 min light adaptation and under 20 cd s m^−2^ of light intensity were recorded by Celeris rodent ERG system. The a‐wave and b‐wave ERG signals, ERG oscillatory potentials (OPs), and PhNR signals were exported from Diagnosis Epsion software.

### Quantitative Analysis of the CNV Area

To confirm the efficacy of neovascular occlusion by the photoresponsive nanosystem, CNV mice with or without treatment were anesthetized. 6 mice with 4 laser injuries per eye per group were used for quantitative analysis of the CNV area. Their eyes were subsequently extracted on day 14. The RPE‐choroid‐sclera complex was harvested after dissecting the cornea, lens, and vitreous body. Afterward, the ocular tissue was stained with CD31 (platelet endothelial cell adhesion molecule, PECAM‐1) antibody (1:200 dilution, MAB1398Z, Millipore, USA) at 4 °C overnight. After being washed with PBS buffer six times and then incubated with secondary antibodies, flat‐mounted RPE‐choroid slides were prepared and the fluorescence images were acquired by confocal microscopy. The CNV lesion area was quantitatively analyzed by ImageJ software.

### Western Blot Analysis of Angiogenic Signaling Biomarkers

After the treatment, choroid tissue was separated and lysed with RIPA buffer containing 1% of protease inhibitor and 1% phosphatase inhibitor for the detection of CD31, phosphorylated Akt and MAPK proteins with Western blot analysis. The protein samples were normalized by BCA protein assay, denatured by being boiled for 10 min, separated by 5%/12% sodium dodecyl sulfate‐polyacrylamide gel electrophoresis (SDS‐PAGE). Then the protein samples were transferred from the gel to nitrocellulose (NC) membrane under constant current at 300 mA for 1.5 h and being incubated by 5% bovine serum albumin (BSA) blocking buffer for 1 h. After that, the protein membranes were incubated with primary antibodies and horseradish peroxidase (HRP)‐secondary antibodies subsequently. After being reacted with the chemiluminescent Western blotting substrate, the protein membranes were visualized by a chemiluminescence imaging system. GAPDH staining was utilized for standardization of the samples. The antibodies and their working dilutions are listed as follows: CD31 antibody (1:500 dilution, GB11063, Servicebio, China), pan‐Akt antibody (1:1000, ab8805, Abcam, UK), pThr308‐Akt antibody (1:1000, ab38449, Abcam, UK), pThr180‐Tyr182‐MAPK antibody (1:1000, AP0526, ABclonal, USA), MAPK antibody (1:1000, A14401, ABclonal, USA), goat anti‐mouse IgG H&L HRP antibody (1:4000, ab6789, Abcam, UK), and goat Anti‐rabbit IgG H&L HRP antibody (1:4000, ab205718, Abcam, UK).

### Histological Assessment

Paraffin‐embedded tissue sections were prepared following the procedures described previously.^[^ ^22^ ^]^ 6 mice with 4 laser injuries per eye per group were used for quantitative analysis of the CNV thickness. Briefly, enucleated eyeballs and the major organs were collected, fixed with FAS eyeball fixative solution, and then embedded in paraffin. Visual axis‐oriented eyeball sections and histological structural oriented organ sections were prepared and then stained with hematoxylin and eosin (H&E) for histologic examination. 50 consecutive cross‐sectioning of each eyeball was performed at an interval of 4 µm near the optic disc to identify the CNV lesions.

### Statistical Analysis

All the data were presented as mean ± SD except for specific notification. Statistical analysis was performed using GraphPad Prism 9.0.1 software. Unpaired two‐tailed student's *t*‐test was used for between‐group comparisons, while one‐way ANOVA test combined with the Dunnett's post hoc test was utilized for multi‐group analysis. Besides, FFA grading results were compared with chi‐square tests. *p* < 0.05 was considered as a statistical significance.

## Conflict of Interest

A PCT application was filed with No. PCT/CN2022/107 621.

## Author Contributions

S.X. and K.C. contributed equally to this work. S. X., K.L., and W.W. conceived the study; X. L. and W.W. supervised the study. S.X. and K.L. synthesized the samples. S.X. and K.C. carried out the experiments and performed the statistical analysis with the help of L.J. N.F. assisted in the LC‐MS/MS analysis. S.X. wrote the manuscript. All authors reviewed and discussed the manuscript and have given approval to the final version.

## Supporting information

Supporting Information

## Data Availability

The data that support the findings of this study are available from the corresponding author upon reasonable request.
