# Correction to “Red Light‐Triggered Anti‐Angiogenic and Photodynamic Combination Therapy of Age‐Related Macular Degeneration”

**DOI:** 10.1002/advs.202416442

**Published:** 2025-01-13

Xu S, Cui K, Long K, Li J, Fan N, Lam W, Liang X, Wang W. Red Light‐Triggered Anti‐Angiogenic and Photodynamic Combination Therapy of Age‐Related Macular Degeneration. *Advanced Science* **2023**, *10*(31), 2301985.

https://doi.org/10.1002/advs.202301985

In Figure 3b, the “Control” and “Control + hv” images were mistakenly chosen from the groups of “VER” and “VER + hv,” respectively, during the layout of the images. The corrected Figure 3b is presented below. As the quantification analysis of the migrated cell area was based on the correct images, the results and conclusions remain unchanged.


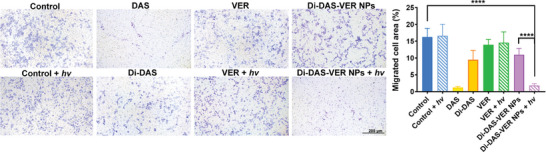


In Figure S9a (Supporting Information), the image of “20 h – VER + hv” was mistakenly duplicated at the position of “20 h – VER” during the layout of the images. The corrected Figure S9 (Supporting Information) is presented below. As the quantification analysis (Figure S9b, Supporting Information) was based on the correct data, the results and conclusions remain unchanged.


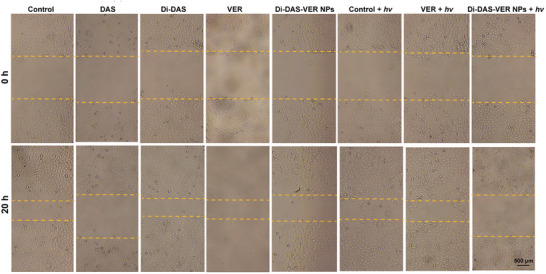


We apologize for these errors.

