# Supplementary material for: Red Light‐Triggered Anti‐Angiogenic and Photodynamic Combination Therapy of Age‐Related Macular Degeneration
Source: Adv Sci (Weinh). 2023 Sep 14;10(31):2301985. doi: 10.1002/advs.202301985 (PMC10625062; doi:10.1002/advs.202301985)
Supplement: Supplementary file 1 — Supporting Information [file ADVS-10-2301985-s001.pdf]

ADVANCED SCIENCE
Open Access

Supporting Information

for *Adv. Sci.*, DOI 10.1002/advs.202301985

Red Light-Triggered Anti-Angiogenic and Photodynamic Combination Therapy of Age-Related Macular Degeneration

*Shuting Xu, Kaixuan Cui, Kaiqi Long, Jia Li, Ni Fan, Wai-Ching Lam, Xiaoling Liang**
*and Weiping Wang**

Supporting information

Red light-triggered anti-angiogenic and photodynamic combination therapy of age-related macular degeneration

Shuting Xu†[1, 2, 3], Kaixuan Cui†[4], Kaiqi Long[1, 2, 3], Jia Li[1, 2, 3], Ni Fan[1, 2, 3], Wai-Ching Lam[5], Xiaoling Liang*[4], Weiping Wang*[1, 2, 3]

1 State Key Laboratory of Pharmaceutical Biotechnology, The University of Hong Kong, Hong Kong, China

2 Department of Pharmacology and Pharmacy, Li Ka Shing Faculty of Medicine, The University of Hong Kong, Hong Kong, China.

3 Laboratory of Molecular Engineering and Nanomedicine, Dr. Li Dak-Sum Research Centre, The University of Hong Kong, Hong Kong, China.

4 State Key Laboratory of Ophthalmology, Zhongshan Ophthalmic Center, Sun Yat-sen University, Guangdong Provincial Key Laboratory of Ophthalmology and Visual Science, Guangzhou 510060, China.

5 Department of Ophthalmology, Vancouver General Hospital, Vancouver, BC V5Z 0A6, Canada

† These authors made equal contributions to this work

*Corresponding author, Prof. X. Liang, E-mail address: liangxlsums@qq.com; Dr. W. Wang, E-mail address: wangwp@hku.hk

RESHULTS

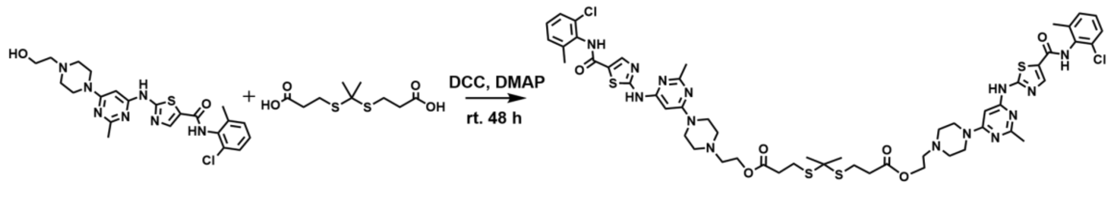

Figure S1. Synthesis route of the dimeric prodrug Di-DAS.

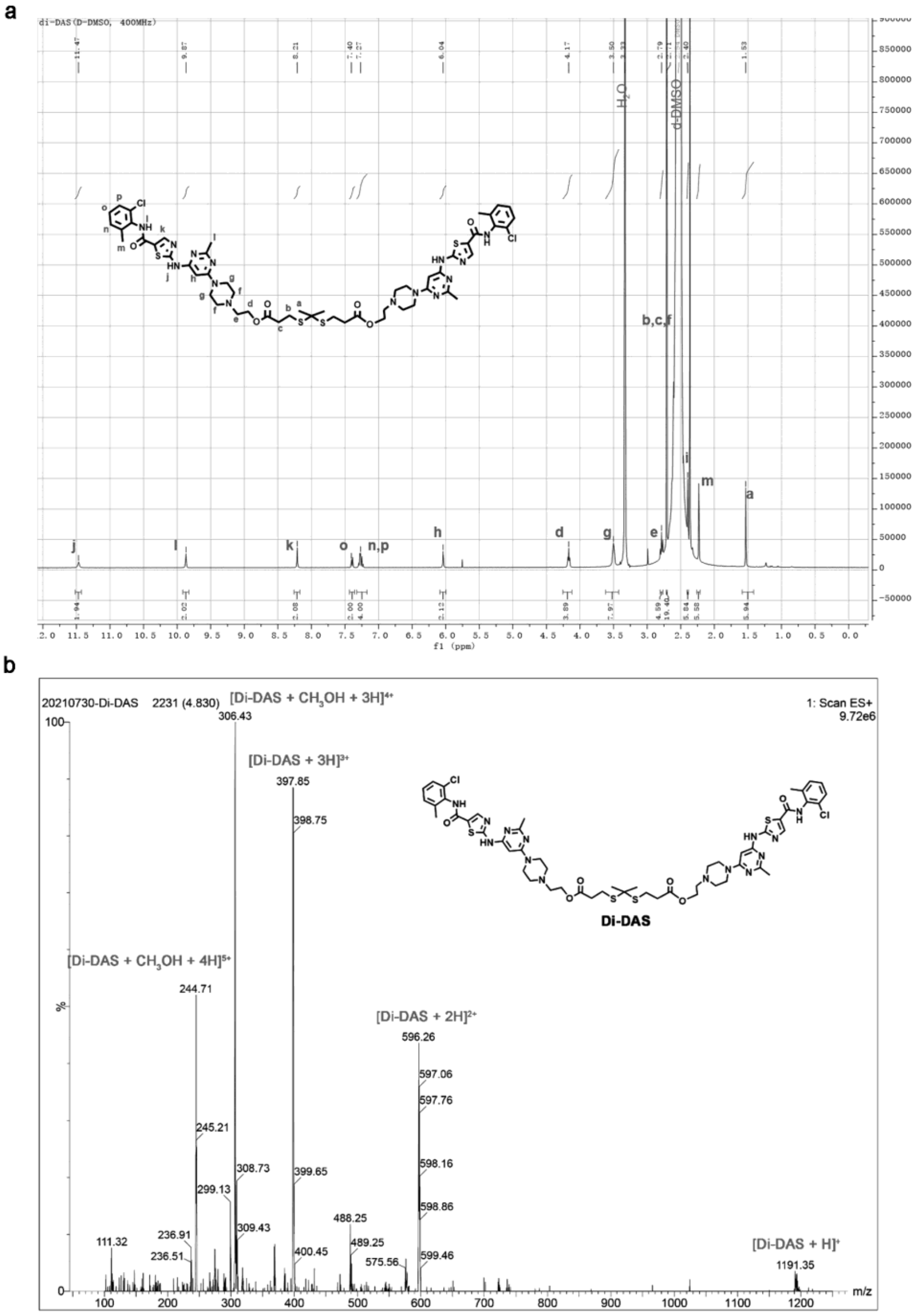

Figure S2. (a) Proton nuclear magnetic resonance spectrum and (b) mass spectrum of Di-DAS.

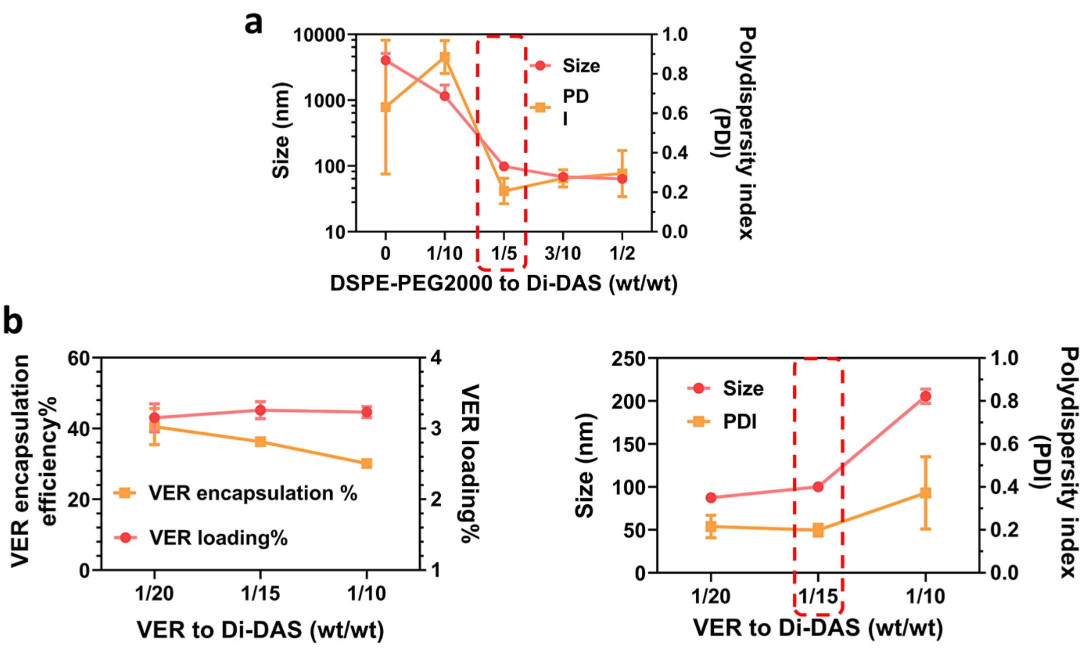

Figure S3. Optimization of Di-DAS-VER NP preparation. (a) Size distribution of Di-DAS-VER NPs with the feeding ratio of 1/20 (VER to Di-DAS, wt/wt) and varying ratios of DSPE-PEG$_{2000}$ to Di-DAS (wt/wt). With the ratio at 1/5, the resulting formulation exhibited the most desirable particle size distribution. (b) VER encapsulation efficiency, VER loading capacity and size distribution of Di-DAS-VER NPs with the feeding ratio of 1/5 (DSPE-PEG to Di-DAS, wt/wt) and varying ratios of VER to Di-DAS (wt/wt). An increase in the feeding ratio of VER did not result in a further increase in VER encapsulation. Particularly, at 1/15 of the feeding ratio of VER to Di-DAS (wt/wt), the resulting formulation exhibited the most desirable particle size distribution. VER: verteporfin; DAS: dasatinib. Data are presented as mean ± standard deviations (n = 3).

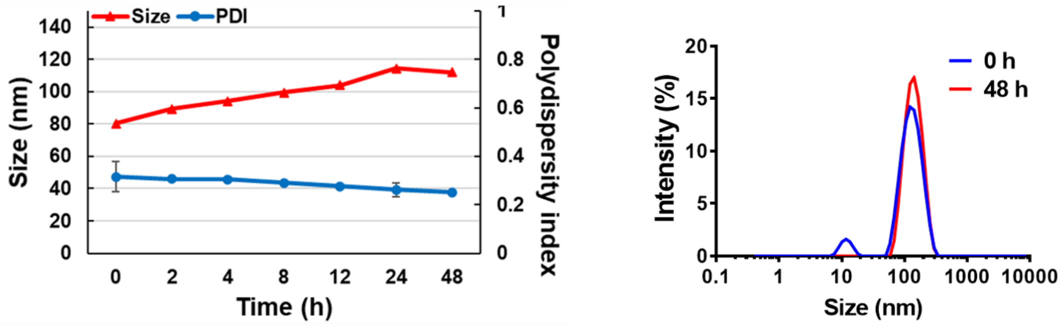

Figure S4. Size distribution of Di-DAS-VER NPs before and after dispersion in DMEM containing 10 % fetal bovine serum (FBS) at 37 °C for 48 h.

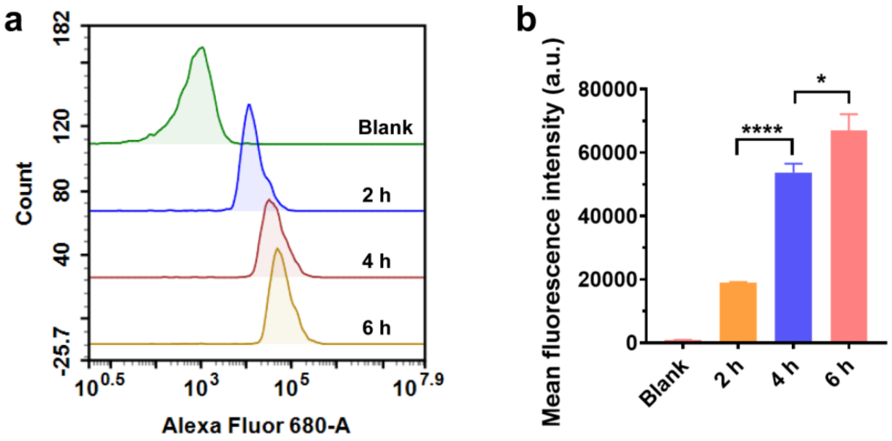

Figure S5. (a) Representative flow cytometry histogram graphs and (b) the corresponding quantification results of HUVEC uptake of Di-DAS-VER NPs after incubation for varying periods. Data are presented as mean ± standard deviations (n = 3). ****: $p < 0.0001$; *: $p < 0.05$.

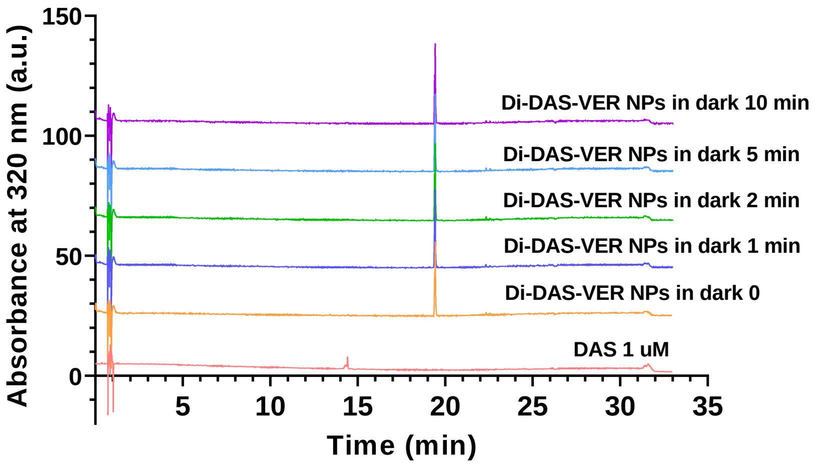

Figure S6. Representative HPLC chromatograms of Di-DAS-VER NPs kept for various time periods without light irradiation.

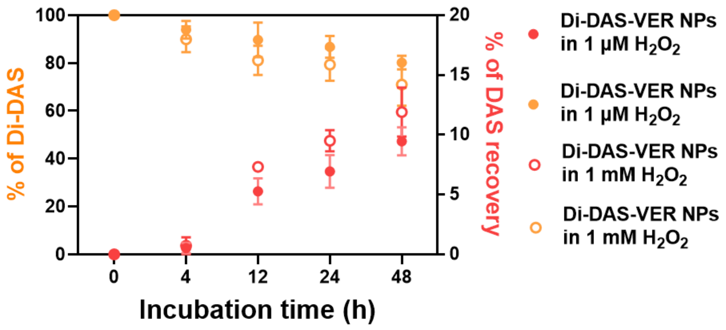

Figure S7. Quantitative analysis of the remaining Di-DAS relative to the initial time point and DAS recovery percentage from Di-DAS-VER NPs after incubation with 1 μM H_2O_2 or 1 mM H_2O_2 for varying periods. Data are presented as mean ± standard deviations (n = 3).

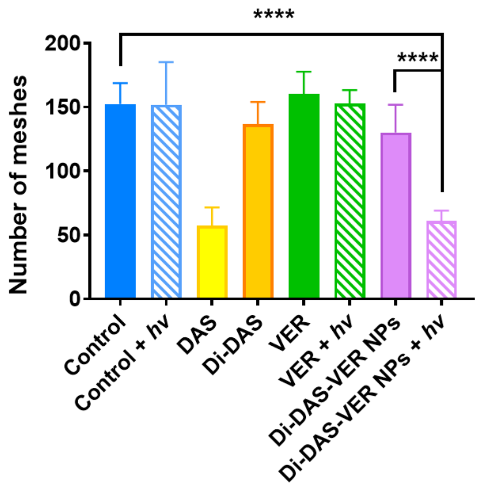

Figure S8. The quantification results of VEGF-stimulated HUVEC tube formation assay of Di-DAS-VER NPs at 4 h post-treatment. HUVEC suspensions were treated with 20 ng/mL $VEGF_{165}$ and co-incubated with different formulations at a DAS equivalent dose of 0.05 μM, followed by 0.75 J/cm^2 of light irradiation at 690 nm or not (10 mW/cm^2, 75 s). Data are presented as mean ± standard deviations (n = 6). ****: $p < 0.0001$.

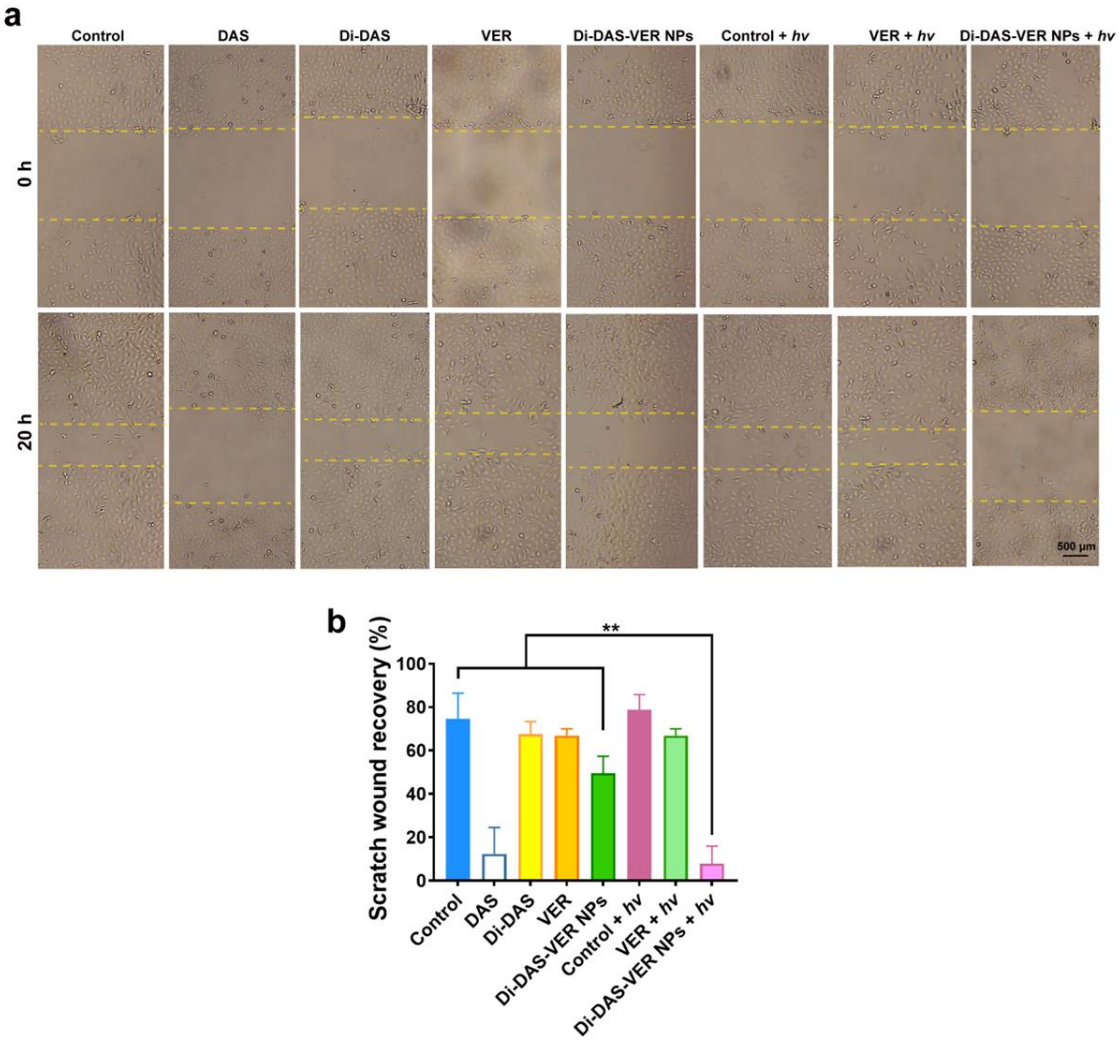

Figure S9. Representative microscopy images (a) and quantitative analysis (b) of HUVEC scratch-wounds at 0 and 20 h after incubation with different formulations with or without 690 nm laser irradiation. Changes of scratch-wound areas were analyzed by ImageJ to calculate the wound recovery rates. DAS, dasatinib; VER, verteporfin. Data were presented as mean ± standard deviations (n = 5 images/group at each time point). **: $p < 0.01$.

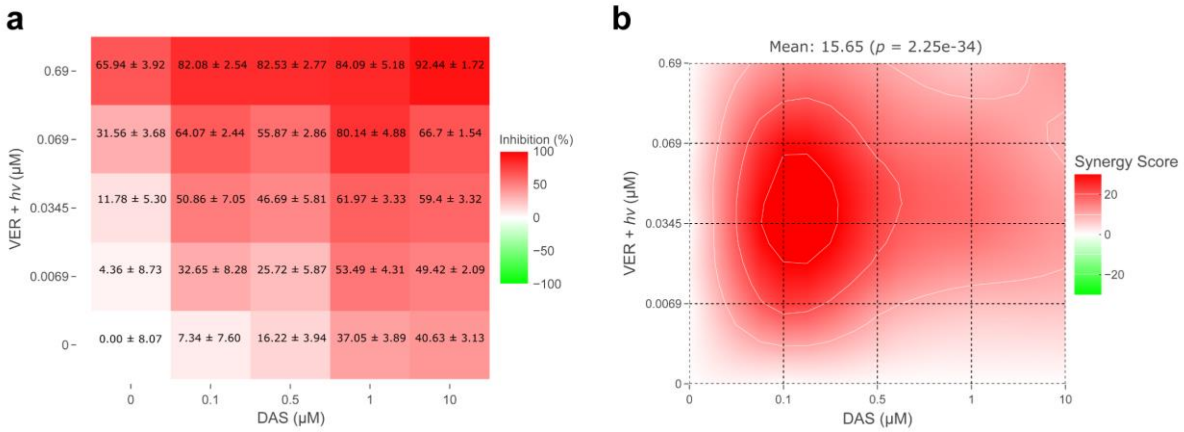

Figure S10. (a) Quantitative analysis of HUVEC cell death percentages after being treated with varying concentrations of DAS and VER plus light irradiation. Cells were treated with 20 ng/mL VEGF165 and cultured with the media containing the indicated drugs. Then cells were exposed to 4 J/cm^2 of light irradiation at 690 nm (10 mW/cm^2, 400 s), and MTT assay was performed at 24 h post-treatment. Data were presented as the mean ± SD (n = 6). (b) Synergy score map corresponding to (a) and calculated by SynergyFinder Plus with Zero interaction potency (ZIP) mode.

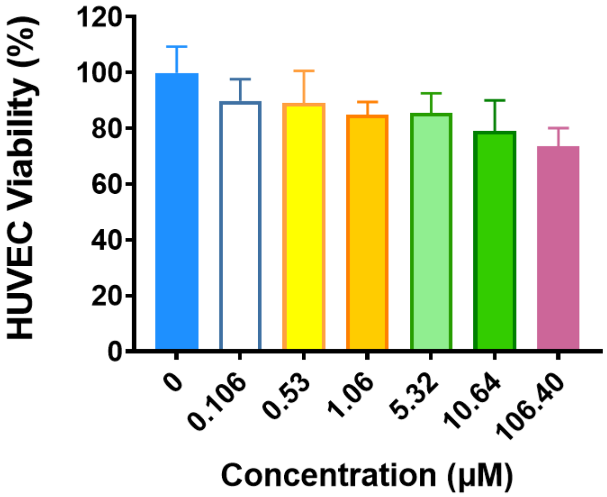

Figure S11. Quantitative HUVEC viability of Di-DAS-VER NP treatment by MTT assay. Cells were incubated with Di-DAS-VER NPs at various concentrations for 48 h. Data were presented as mean ± standard deviations (n = 6).

Table S1. Pharmacokinetic parameters of different formulations after tail vein injection in C57BL/6 mice.

Parameter	DAS	VER	Di-DAS-VER NPs
$t_{1/2}$ (h)	2.808 ± 0.522	4.996 ± 0.039	6.100 ± 0.1277
C_{max} (ng/mL)	6186.753 ± 314.953	1346.773 ± 4.720	5869.108 ± 428.550
$AUC_{0\text{-}t}$ (ng·h/mL)	5020.356 ± 999.812	4875.461 ± 110.528	16968.201 ± 1061.769
$MRT_{0\text{-}inf\_obs}$ (h)	2.760 ± 0.624	7.169 ± 0.007	8.309 ± 0.195
Vz_{obs} (L/kg)	34.976 ± 7.146	1.941 ± 0.066	16.380 ± 1.162
Cl_{obs} (L/h/kg)	8.602 ± 0.165	0.269 ± 0.007	1.859 ± 0.929

Note: $t_{1/2}$, plasma terminal elimination half-life; C_{max}, maximum observed drug concentration; $AUC_{0\text{-}t}$, area under the drug concentration-time curve from time 0 to time t; $MRT_{0\text{-}inf\_obs}$, apparent mean residence time of drug; Vz_{obs}, apparent volume of drug distribution; Cl_{obs}, apparent total body clearance of the drug from the blood. Data were analyzed by PKSolver with noncompartmental data analysis mode, and presented as mean ± SD (n =3). DAS, dasatinib; VER, verteporfin.

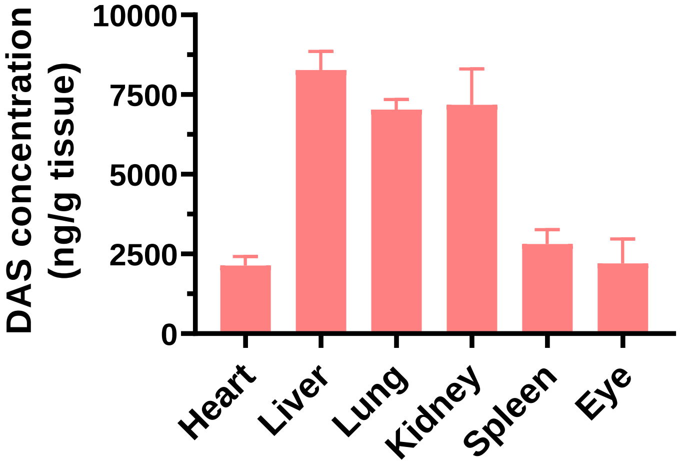

Figure S12. HPLC quantitative analysis of DAS concentration at 2 h post-intravenous administration of DAS solution in experimental CNV mice. Data were presented as mean ± standard deviations (n = 3).

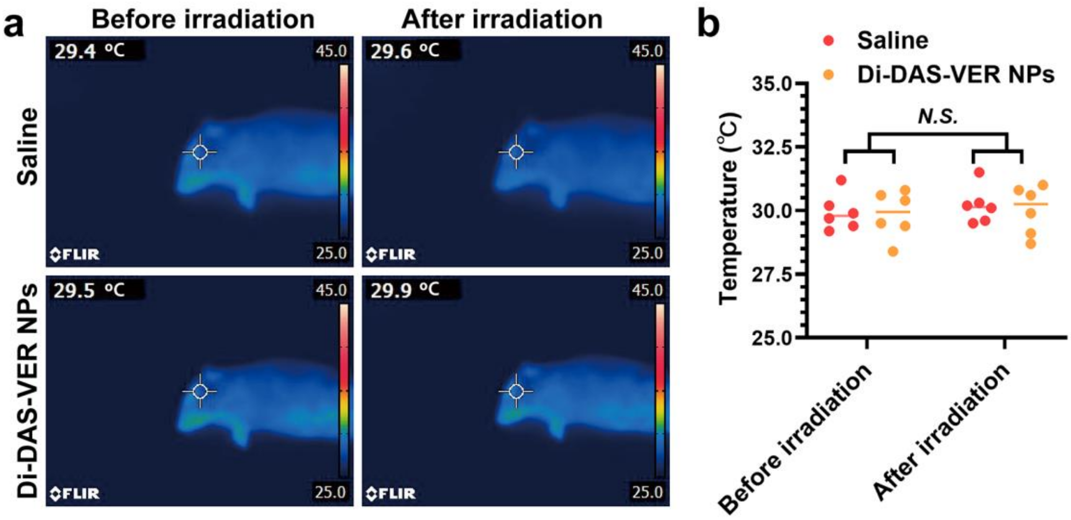

Figure S13. (a) Representative infrared thermal images and (b) temperature variations of the mouse eyes before and after the indicated treatments with 690 nm light irradiation. Data were presented as mean ± standard deviations (n = 6). *N.S.*: No statistical significance.

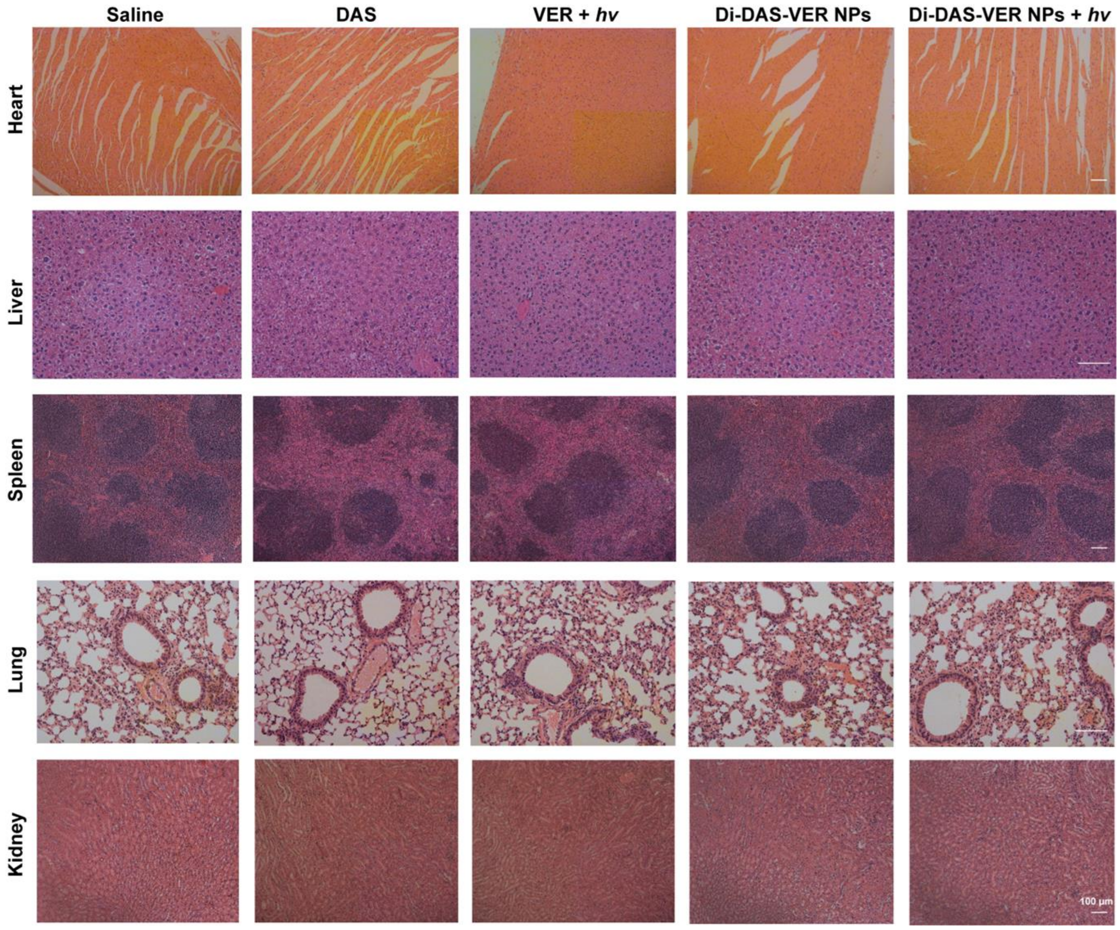

Figure S14. Representative photomicrographs of H&E-stained main organ sections of the mice after treatment with different formulations. DAS, dasatinib; VER, verteporfin.
